# Molecular mechanism of co-transcriptional H3K36 methylation by SETD2

**DOI:** 10.1038/s41467-025-65439-y

**Published:** 2025-10-29

**Authors:** James L. Walshe, Moritz Ochmann, Ute Neef, Olexandr Dybkov, Christian Dienemann, Christiane Oberthür, Aiturgan Zheenbekova, Henning Urlaub, Patrick Cramer

**Affiliations:** 1 Department of Molecular Biology, Max Planck Institute for Multidisciplinary Sciences, Göttingen, Germany; 2 Bioanalytical Mass Spectrometry, Max Planck Institute for Multidisciplinary Sciences, Göttingen, Germany; 3 Institute of Clinical Chemistry, Bioanalytics Group, University Medical Center Göttingen, Göttingen, Germany

**Keywords:** DNA methylation, Cryoelectron microscopy, Histone post-translational modifications, Transcription

## Abstract

H3K36me3 is a hallmark of actively and recently transcribed genes and contributes to cellular memory and identity. The deposition of H3K36me3 occurs co-transcriptionally when the methyltransferase SETD2 associates with RNA polymerase II. Here we present three cryo-EM structures of SETD2 bound to RNA polymerase II elongation complexes at different states of nucleosome passage. Together with functional probing, our results suggest a 3-step mechanism of transcription-coupled H3K36me3 deposition. First, binding to the elongation factor SPT6 tethers the catalytic SET domain in proximity to the upstream DNA. Second, RNA polymerase II nucleosome passage leads to the transfer of a hexasome from downstream to upstream, poised for methylation. Finally, continued transcription leads to upstream nucleosome reassembly, partial dissociation of the histone chaperone FACT and sequential methylation of both H3 tails, completing H3K36me3 deposition of an upstream nucleosome after RNA polymerase II passage.

## Abstract (web summary)

H3K36me3 is a marker of actively and recently transcribed genes. Here, the authors describe a 3-step mechanism of transcription-coupled H3K36me3 deposition by the mammalian methyltransferase SETD2, via the elongation factor SPT6.

## Introduction

During transcription of a gene, RNA polymerase (Pol) II progresses rapidly through regular arrays of nucleosomes. Despite the large-scale movements associated with chromatin transcription, Pol II passage through a nucleosome generally involves transfer of the nucleosome from incoming DNA located in front of Pol II (downstream) to DNA in the wake of Pol II (upstream). Such nucleosome transfer during Pol II passage prevents the loss of information contained in the form of covalent histone modifications^1–5^.

The process of Pol II nucleosome passage requires the concerted action of several elongation factors, chromatin remodelers and histone chaperones^6–10^. Elongation factors associated with activated elongating Pol II include DSIF (SPT4/5), SPT6, TFIIS, and PAF1c (complex of CTR9, PAF1, LEO1, WDR61, CDC73, and RTF1)^10–12^. In vivo, SPT6 has the strongest influence on Pol II processivity and has histone chaperone activity^13,14^. The PAF1c subunit RTF1 has a profound impact on the Pol II elongation rate in vitro^7,10,15^ and is implicated in the maintenance of chromatin by recruiting histone remodelers and modifiers^15–22^. In addition to these elongation factors, the histone chaperone FACT (a heterodimer of subunits SPT16 and SSRP1) stimulates chromatin transcription^6,8,23–26^ and is also implicated in chromatin maintenance^27–34^. Recently, cryo-EM structures of the yeast activated elongation complex transcribing through a nucleosome have progressed our understanding of the mechanisms underlying Pol II chromatin passage^6,28,35,36^.

However, despite this progress, it remains unknown how Pol II nucleosome passage is coupled to co-transcriptional histone modification. One of the most prominent histone modifications acquired during Pol II transcription is the tri-methylation of histone H3 on residue lysine-36 (H3K36me3) that was first described over 20 years ago^37–42^. H3K36me3 acts as docking sites for various chromatin readers, including PWWP, chromo- and tudor domains^43–46^ and is implicated in pre-mRNA processing, DNA mismatch repair^47^ and chromatin integrity^48^. Changes in H3K36me3 levels lead to deleterious intergenic transcription that is associated with cancerous cell growth^49,50^.

The deposition of H3K36me3 is carried out by the conserved histone methyltransferase SETD2^51^. SETD2 associates with Pol II through an interaction of its Set2 RPB1-interaction (SRI) domain with the phosphorylated C-terminal domain (CTD) of the largest Pol II subunit RPB1^40,52,53^. The SRI domain also regulates SETD2 activity by antagonizing the effect of the SET auto-inhibitory domain (AID)^54^. The yeast homolog of SETD2, Set2, interacts genetically with SPT6^55,56^. To date, structural analysis of SETD2 is limited to individual domains^52,53,57^ and the catalytic SET domain bound to a nucleosome^54^.

In this study, we investigate the molecular-mechanistic basis of co-transcriptional H3K36me3 deposition by SETD2. We demonstrate H3K36me3 deposition occurs in the wake of elongating Pol II, after the nucleosome has been transferred to upstream DNA. We present three cryo-EM structures of the activated Pol II elongation complex in the presence of SETD2 and FACT and probe these structures with functional assays. Our structure-function analysis provides a model for a 3-step molecular mechanism of co-transcriptional H3K36me3 deposition.

## Results

### H3K36me3 deposition requires Pol II nucleosome passage

**Fig. 1:**
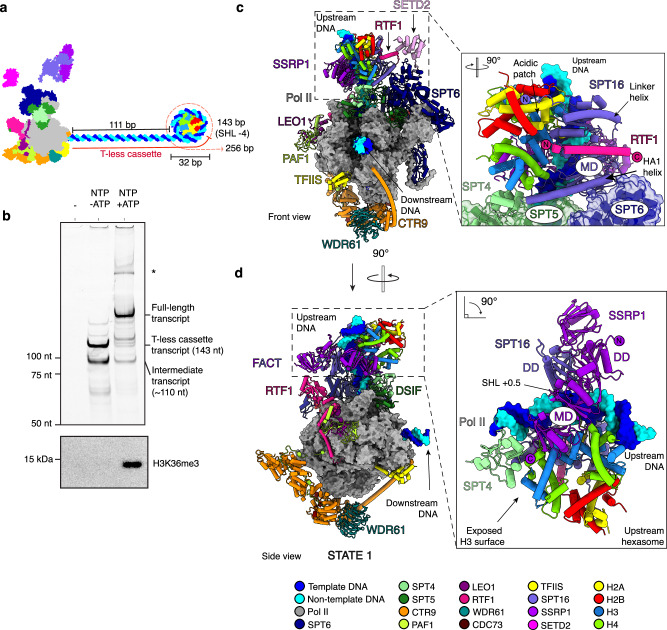
Upstream H3K36me3 activity and cryo-EM structure of the State 1 poised methylation complex. **a** Schematic representation of nucleosome template and proteins used in (**b**) SETD2 deposits H3K36me3 on an upstream nucleosome. RNA extension by the methylation-competent elongation complex through mononucleosomes with extended run-up distance and T-less cassette to bp 32 (SHL − 4) into the nucleosome. RNA primer contains a 5’ Cy5 label. RNA lengths are indicated on the right side of the denaturing PAGE gel and ssRNA marker on the left. Western blot for H3K36me3 of RNA extension assay products. Assay performed in triplicates. Source data are provided as a Source Data file. **c** Overall structure of the State 1 complex with magnified view of FACT binding to upstream hexasome. **d** Alternate view of the State 1complex.

To investigate the mechanism underlying co-transcriptional H3K36me3 deposition, we first determined whether SETD2 methylates the nucleosome downstream or upstream of the transcribing Pol II. To this end, we performed RNA extension assays on a DNA template wrapped around a single nucleosome positioned 111 bp downstream from the extension start site and monitored H3K36me3 deposition with qualitative Western blot analysis. A T-less cassette was used to enable stalling of Pol II at base pair (bp) 32 of a modified Widom 601 sequence (Fig. 1a). Poll II stalled at this position would unwrap DNA to at least superhelical location (SHL) − 4, providing an optimal downstream nucleosome substrate for H3K36me3 deposition^58^.

The complete mammalian activated elongation complex^10^ was assembled on the nucleosomal DNA construct and the factors TFIIS, FACT, IWS1 and SETD2 (residues 1435–2567) were added. H3K36me3 deposition was analyzed 10 min after nucleotide addition (Fig. 1b). No H3K36me3 was detected in the absence of transcription, corroborating that methylation occurs co-transcriptionally in our minimal biochemical system. Also, H3K36me3 was absent after transcription was stalled at bp 143 (32 bp into the nucleosome), when Pol II is located in front of the incoming nucleosome. A signal for H3K36me3 was only observed after transcription had occurred to the end of the template (bp 256). This suggests that co-transcriptional H3K36me3 deposition occurs on the upstream nucleosome after Pol II passage (Fig. 1b).

### Architecture of the poised methylation complex

In order to investigate how SETD2 methylates an upstream nucleosome, we biochemically and structurally analyzed the progression of the complete mammalian activated elongation complex^10^ in the presence of TFIIS, FACT, IWS1 and SETD2. We stalled the complex at a position that is known to trigger nucleosome transfer upstream of the Pol II (bp 64 of a modified Widom 601 sequence)^28^. The complex was mildly crosslinked and purified in a grafix gradient. Fractions containing the stalled complex (called here methylation-competent elongation complex) were used for single-particle cryo-EM analysis (Supplementary Fig. 1). We obtained a reconstruction that showed Pol II at a resolution of 2.85 Å (Supplementary Fig. 2). Focused classification of density upstream of Pol II revealed a FACT-bound hexasome and SETD2 bound to SPT6. An overall model for the poised methylation complex (State 1), was built using the obtained density maps (Supplementary Fig. 2, 3), data from chemical crosslinking-mass spectrometry (CXL-MS) (Supplementary Fig. 4a, b and Supplementary Data 1) and Alphafold2 or ColabFold predictions^59,60^ (Supplementary Fig. 5) (Supplementary Table 1).

In State 1, FACT binds the transferred hexasome in the region of upstream DNA above the Pol II active center cleft (Fig. 1c, d). Relative to the direction of transcription, the hexasome is missing the proximal H2A–H2B dimer and is consistent with a similar state captured for the yeast Pol II^28^. Approximately 20 bp of DNA covers the histones from SHL − 0.5 to SHL + 1.5 (Fig. 1d). FACT is bound to all histones and the DNA, covering a total of ~5200 Å^2^ in buried surface area. Its dimerization domains (DD) interact with the nucleosomal DNA at SHL + 0.5, ~30 bp upstream of the Pol II active center (Fig. 1d). The middle domain (MD) of SPT16, that includes the HA1 helix (residues 759–791), contacts both the (H3–H4)_2_ tetramer and the distal H2A-H2B dimer (Fig. 1c and Supplementary Fig. 4e). The SPT16 linker helix (residues 468–498) extends towards the acidic patch of the H2A–H2B dimer (Fig. 1c) and the residues of the C-terminal domain (CTD) bind the exposed surface of the H2A–H2B dimer (Supplementary Fig. 4d, e) as described^28,61–63^. The SSRP1 middle domain (MD) contacts both the (H3–H4)_2_ tetramer and DNA on the opposite side to SPT16 (Fig. 1d). Together, these interactions anchor FACT to the hexasome.

**Fig. 2:**
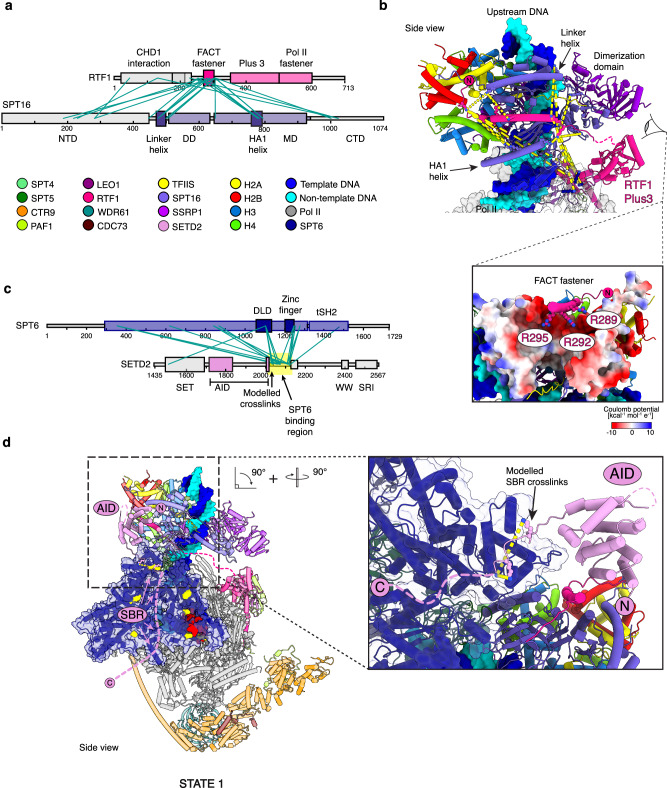
CXL-MS analysis poised methylation complex. **a** Schematic overview of the RTF1 and SPT16 domain architecture. Interchain BS3 crosslinks are visualized between RTF1 and SPT16. Only crosslinks with a -log_10_Score > 1 are shown. Crosslinks to the N-terminal remnant of the RTF1 and SPT16 affinity tags are not displayed. Initial visualization of crosslinks performed in xiNET^100^. **b** Magnified view of FACT “fastener” binding SPT16. Interchain BS3 crosslinks are visualized. SPT16 visualized as coulombic surface potential. **c** Schematic overview of the SPT6 and SETD2 domain architecture. Interchain BS3 crosslinks are visualized between the proteins (**d**) overall structure of the complex. SPT6 residues that crosslink with SETD2 colored yellow. Magnified view of the SPT6 binding region of SETD2.

Elongation factors DSIF (SPT4/SPT5) and SPT6 are well positioned to stabilize the FACT-bound upstream hexasome. SPT5 is in close proximity to the SPT16 MD and the HA1 helix (Fig. 1c). SPT4 stabilizes the exposed surface of histone H3 and the C-terminal end of the SSRP1 MD (Fig. 1d). Compared to the canonical complete activated elongation complex^10^, SPT6 rotates towards the upstream DNA by ~ 20° (Supplementary Fig. 4f) providing an additional contact point between the elongation complex and the hexasome (Fig. 1c). The absence of this contact coincides with badly resolved nucleosome density (Supplementary Fig. 2c) and indicates stabilization of the hexasome by SPT6. This is consistent with the established role for SPT6 as a chaperone^13,14,64,65^. Based on CXL-MS data (Fig. 2a, b), we placed residues 266–315 of RTF1 into helical density on the surface of SPT16. This helix is conserved in higher eukaryotes (Supplementary Fig. 6) with basic residues Arg 289, Arg 292 and Arg 295 well positioned to bind to the acidic surface of the middle domain of SPT16 (Fig. 2b and Supplementary Fig. 4c). Due to the direct interaction between RTF1 and FACT, we termed the RTF1 helix the FACT “fastener”. Collectively, we have established in the mammalian context that both SPT6 and FACT fulfill roles as histone chaperones by stabilizing a newly transferred hexasome in the wake of Pol II transcription. In addition, elongation factors DSIF and RTF1 stabilize the interactions between FACT and the hexasome, demonstrating the intrinsic link between Pol II elongation and nucleosome retention.

### SETD2 binds to SPT6

Careful inspection of our cryo-EM maps revealed density in proximity of SPT6 and the upstream hexasome that could not be assigned to a known feature. Further classification on signal-subtracted particles containing only SPT6 showed extra density on the death-like domain (DLD) surface (Supplementary Figs. 3e, 7a). This density could be assigned to a conserved 7-residue stretch of SETD2 (Supplementary Fig. 8) based on our CXL-MS data and Colabfold^59^ predictions (Fig. 2c, d and Supplementary Fig. 5b). It is located at the beginning of an unstructured region of SETD2 (residues 2022–2130) that strongly crosslinks to SPT6 and was named the SPT6 binding region (SBR) (Fig. 2c, d). Only 14 residues of this region could be modeled into our observed density, suggesting the remaining residues are flexible.

The SBR of SETD2 is located C-terminal of the SETD2 auto-inhibitory domain (AID) (Fig. 2c). At low threshold and with a gaussian filter applied, we observed globular density into which the Alphafold2^60^ model of the SETD2 AID domain could be fitted (Supplementary Fig. 3e, 7b). The AID domain is located between the FACT “fastener” of RTF1 and the DLD of SPT6 (Figs. 1c, 2d and Supplementary Fig. 7b). We did not observe density for the catalytic SET domain, but since it immediately follows the AID (Fig. 2c), the catalytic SET domain is located in proximity to an upstream nucleosome during reassembly. In State 1, the catalytic SET domain is occluded from binding the hexasome by the presence of FACT. In summary, State1 shows the SBR tethers the AID and catalytic SET domain to SPT6 in proximity to the transferred hexasome, poised for H3K36me3 deposition upon FACT dissociation from the nucleosome dyad.

### Architecture of the proximal H3K36me3 writer complex

Having identified a poised state for H3K36me3 deposition, we investigated how SETD2 would methylate an intact upstream nucleosome. We reconstituted the methylation-competent elongation complex on a ligated nucleosome template that would represent transcription to bp 159 of a Widom 601 sequence, such that the nucleosome is located upstream of Pol II. The complex was purified by size exclusion chromatography and a single peak fraction containing the complex were crosslinked, quenched and flash-cooled for single particle cryo-EM analysis (Supplementary Fig. 9a). The obtained data yielded a reconstruction that revealed Pol II at a resolution of 2.63 Å. Focused 3D classification of the upstream region resulted in a 4.8 Å reconstruction of the catalytic SET domain (residues 1452–1696) bound to the nucleosome that we refer to as State 2 (Fig. 4b, e, Supplementary Figs. 10, 11 and Supplementary Table 2).

**Fig. 3:**
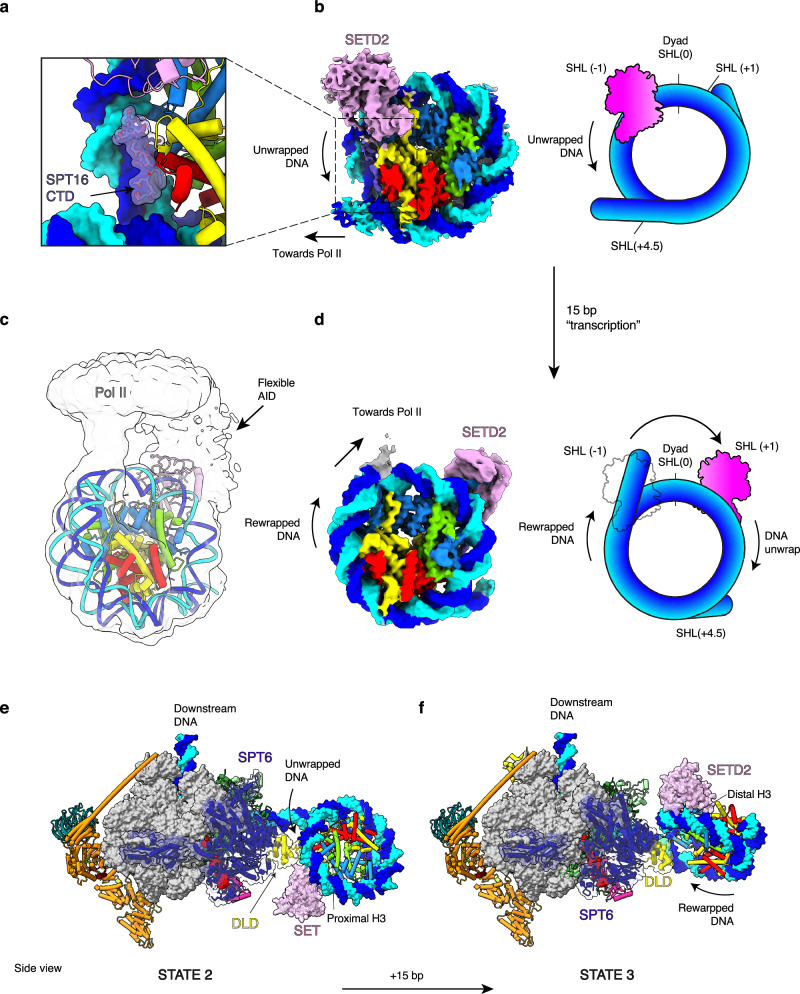
Cryo-EM structure of SETD2 bound to proximal and distal H3 tail. **a** SPT16 CTD binds exposed H2A–H2B dimer of unwrapped nucleosome. **b** 4.84 Å reconstruction of SETD2 bound to proximal H3 tail of the unwrapped upstream nucleosome (**c**), 3.6 Å reconstruction of SETD2 bound to the distal H3 tail of the unwrapped upstream nucleosome. A gaussian filter with a 2σ standard deviation was applied to the cryo-EM map. **d** As in (**c**), without filter (**e**, **f**), overall architecture of State 2 and State 3 complexes with SETD2 bound to the proximal and distal H3 tails, of an upstream nucleosome, respectively.

Compared to the upstream hexasome observed in State 1, in State 2 the catalytic SET domain now binds to the proximal H3 tail at SHL − 1 (Fig. 3b). In contrast to previous structures of catalytic SET domains bound to nucleosomes^58,66^, in State 2 the CTD of SPT16 (residues 937-951) stabilizes the SETD2-bound partially unwrapped nucleosome (Fig. 3a, b). Although this region of SPT16 is known to bind free H2A–H2B dimers^61,67^, we observe this interaction in the context of a complete nucleosome. This interaction represents a late stage of nucleosome reassembly after FACT deposits the remaining H2A–H2B dimer, prior to complete dissociation. This suggests an additional role for FACT in facilitating H3K36me3 deposition, apart from its known role in stimulating chromatin transcription and maintenance.

Applying a Gaussian filter to the nucleosome reconstruction allowed further tracing of the DNA, providing an estimate of Pol II direction, and confirming SETD2 is bound to the proximal H3 histone (relative to Pol II) (Supplementary Fig. 12a, b). Surprisingly, adjacent to the catalytic SET domain, above SHL − 1, we identified globular density in our cryo-EM map (Supplementary Fig. 12b). There are only 20 unstructured amino acids between the last modeled residue of the SET domain and the beginning of the helical AID, therefore we assumed this density corresponds to the AID. Consistent with this assumption, the Alphafold2^60^ prediction of the AID fits within the additional density with only 16 Å between the connecting residues (Supplementary Fig. 12a, b). In this conformation, the SBR of SETD2 is directed towards Pol II and SPT6 (Supplementary Fig. 12c).

To accurately determine the orientation of the upstream nucleosome relative to Pol II, multibody refinement in RELION^68^ was performed on the subset of Pol II particles that contained well-aligned nucleosomes (Supplementary Fig. 13a). Given the high flexibility, further classification was performed based on the first principal motion competent (Supplementary Fig. 13b). Refinement of selected particles led to a 12.4 Å reconstruction that allowed faithful positioning of both nucleosome and Pol II models (Fig. 3e and Supplementary Fig. 13c). Although the AID remains highly flexible, the C-terminus of the catalytic SET domain is orientated towards the DLD of SPT6 and indicates the AID and SBR of SETD2 likely remain tethered to SPT6 (Fig. 3e and Supplementary Fig. 12b, c). Compared to State 1, the overall architecture of State 2 demonstrates that continued transcription would reassemble and position the nucleosome at the upstream edge of Pol II. FACT dissociates from the nucleosome dyad but remains loosely tethered to stabilize the partially unwrapped nucleosome state. SETD2 is tethered to SPT6 such that the AID does not prevent binding of the catalytic SET domain to the proximal H3 tail of nucleosome.

### Structure of the distal H3K36me3 writer complex

To methylate both H3 tails of an upstream nucleosome, SETD2 would need to access both the proximal and the distal H3 histone tails during transcription. Re-wrapping of the nucleosome observed in State 2 would rotate the nucleosome, with respect to Pol II, such that the distal H3 histone would be in proximity to SPT6. To investigate this, we prepared another complex for cryo-EM analysis on a 15 bp longer ligated nucleosome template, representing transcription to bp 174 into a Widom 601 sequence. Sample preparation, data collection and processing were performed similarly as for the State 2 complex (Supplementary Figs. 9b, 14–16), leading to a reconstruction with a resolution of 2.6 Å for Pol II and 3.6 Å reconstruction of SETD2 bound to the nucleosome.

In the resulting State 3 structure, SETD2 is bound to the distal H3 tail at SHL + 1 (Fig. 3c, d and Supplementary Table 3). The additional 15 bp of DNA induced approximately 20 bp of DNA to rewrap the nucleosome, making the proximal H3 tail inaccessible for SETD2 binding (Fig. 3c, d). SETD2 binding to the distal H3 tail, at SHL + 1, facilitated unwrapping of distal DNA to approximately SHL + 4.5. Analysis of the overall architecture of the distal H3K36me3 complex demonstrates the nucleosome rewrapping has rotated the nucleosome by ~ 45°, positioning SETD2 on the opposite side of SPT6 (Fig. 3f), such that the interaction between SETD2 and SPT6 could be maintained. Compared to State 2, the overall architecture of State 3 demonstrates that continued transcription would allow rewrapping of the nucleosome that repositions the distal H3 tail of the nucleosome to allow the catalytic SET domain to bind, whilst maintaining an interaction with SPT6.

### SETD2-SPT6 interaction is required for H3K36me3 deposition

**Fig. 4:**
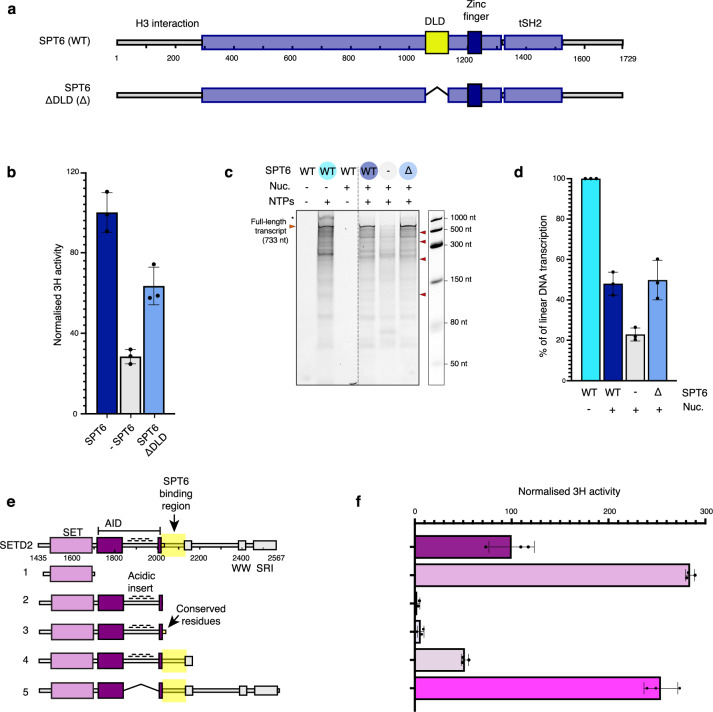
SPT6 SETD2 interaction required for efficient methylation. **a** Schematic overview of the SPT6 showing DLD truncation. **b** Normalized 3H activity of co-transcriptional methylation assays. **c** Denaturing PAGE gel of co-transcriptional methylation assays as in (**b**), with cold S-Adenosyl methionine. Expected nucleosome dyad positions indicated on the right. Undigested DNA/RNA hybrid indicated with *. **d** Quantification of assay replicates in (**c**). **e** Schematic overview of the SETD2 truncations (**f**), normalized 3H activity of co-transcriptional methylation assays performed with SETD2 or truncations. For data presented in (**b**, **f**), each point reflects one replicate (*N* = 3), depicted as mean ± s.d. Source data are provided as a Source Data file.

In our structures, we observed 7 conserved residues of the SBR that bind to the DLD of SPT6. To tested the functional importance of the DLD domain we performed co-transcriptional methylation assays using a SPT6 ΔDLD construct (Fig. 4a). In these assays we used a chromatinized template consisting of four Widom 601 positioned nucleosomes separated by 30 bp of linker DNA. Omitting SPT6 from the reaction resulted in a ~75% decrease in methylation activity, whereas the deletion of the DLD resulted in a ~ 40% decrease (Fig. 4b). Although these results suggest a role for the DLD in SETD2-dependent histone H3 methylation, SPT6 is an elongation factor and may indirectly facilitate H3K36me3 deposition by increasing transcription that exposes the SETD2 binding sites at SHL +/– 1. To test this possibility, transcription activity of elongation complexes formed with full-length SPT6 and ΔDLD mutant were analyzed by denaturing PAGE. Extension of longer tetra-nucleosome templates (Fig. 4c) resulted in increased incomplete transcripts compared to shorter single Widom 601 sequences (Fig. 1b), suggesting increased Pol II drop-off. The decrease in methylation in the absence of full-length SPT6 is likely due, in part, to decreased transcription (Fig. 4c, d), however, no change in transcription was observed for the SPT6 ΔDLD, indicating the methylation deficit was caused by the absence of the DLD. These results disentangle SPT6-stimulated transcription from co-transcriptional H3K36me3 deposition, and conclusively show that the SPT6 DLD is required for efficient co-transcriptional H3K36me3 deposition.

To further test the functional importance of the SETD2 SBR, we designed a series of SETD2 truncations (Fig. 4e) and performed further co-transcriptional methylation assays. As a positive control, we included a SETD2 truncation containing only the catalytic SET domain without auto-regulatory elements. As expected, the catalytic SET domain alone (Fig. 4f Construct 1) showed almost three-fold higher methylation levels than WT (Fig. 4f). The addition of the AID abolished methylation (Fig. 4f Construct 2), consistent with its described role in yeast^54^. Surprisingly, a construct that contained the conserved region of the SBR that could be modeled in our cryo-EM density showed no increase in methylation (Fig. 4f Construct 3). A longer SETD2 variant that includes the complete SBR (Fig. 4f Construct 4) recovered methylation to approximately ~ 50% compared to WT levels, consistent with our crosslinking data and highlights the importance of the full SBR for SETD2 activity. Complete recovery of methylation was not observed as Construct 4 lacks the SRI domain that has been implicated in both Set2 recruitment to chromatin and activation^54,56^. These results strongly support the model that binding of SETD2 to SPT6 via the SBR-DLD interaction is important for high-efficiency H3K36me3 deposition.

### Metazoan-specific acidic insertion in SETD2 auto-inhibitory domain regulates nucleosome binding

Although only 7 residues are conserved between the yeast and human SBR (Supplementary Fig. 17a), the complete SBR is required to partially alleviate SETD2 auto-inhibition (Fig. 4e, f, Construct 4). This indicates an additional metazoan-specific regulatory mechanism must exist. Inspection of the AlphaFold2 predictions between yeast and human H3K36 methyltransferases identified a 170 amino acid insertion within the AID of SETD2. Removal of this insertion dramatically increased SETD2 activity in our co-transcriptional methylation assays (Fig. 4f Construct 5), similar to that of the unregulated SET domain alone. This confirms this region to be important for the regulation of SETD2 activity.

Analysis of the primary sequence revealed this insertion to be highly acidic (pI 4.2) and contained numerous putative phosphorylation sites (Supplementary Fig. 8)^69^. We speculated the acidic insertion may be competing with the nucleosome for binding of the SET domain. Indeed, electromobility shift assays on reconstituted nucleosomes showed tighter binding when the acidic insertion was removed (Supplementary Fig. 17c). To confirm this competition, analytical gel filtration demonstrated a direct interaction between the AID and the SET domain, that could be disrupted by increasing ionic strength (Supplementary Fig. 17d). Taken together, these results identify a regulatory element, within the metazoan SETD2 AID, that is critical for the regulation of SETD2 activity.

## Discussion

To enable co-transcriptional H3K36me3 deposition while preventing spurious methylation events, SETD2 activity is tightly regulated in cells^54,70,71^. In the absence of transcription, the inhibitory effect of the SETD2 AID prevents spurious H3K36me3 deposition on unwrapped nucleosomes that are generated by DNA replication or repair^54^. SETD2 recruitment to chromatin involves an interaction between the SETD2 SRI and the RPB1 CTD^37^, but the molecular mechanism to overcome auto-inhibition remained unclear. Here, we provide three cryo-EM structures and complementary biochemical data that provide answers to these open questions and expand our understanding of how SETD2 is bound to an activated Pol II elongation complex and how it is positioned for co-transcriptional H3K36me3 deposition.

With respect to the binding of SETD2 to the transcribing polymerase complex, we have identified a SPT6 binding region within SETD2 that positions the catalytic SET domain at the upstream edge of Pol II. The direct SPT6 interaction explains the genetic interactions previously observed^55^ and the incomplete depletion of Set2 from chromatin when the SRI domain is removed^56^. In yeast, when only the catalytic SET domain was fused to the CTD of RPB1, no H3K36me3 was detected and indicates the SBR is critical for the correct positioning of the catalytic SET domain at the upstream edge of Pol II. In addition, we identified a metazoan-specific regulatory element within the AID of SETD2 that strongly inhibits methylation by competing with the transcribed nucleosome for binding to the catalytic SET domain. We speculate this region may be used to regulate the recently reported non-catalytic functions of SETD2^72^. When tethered to SPT6, the AID of SETD2 is unable to inhibit binding of the catalytic SET domain to a nucleosome that reassembles in the wake of Pol II transcription. In summary, this structure-function analysis of SETD2 shows both the SRI domain and the SBR are critical for regulating co-transcriptional H3K36me3 deposition. In light of these results, our data suggests a simple mechanism that physically couples SETD2 binding to the activated Pol II elongation complex to its functional activation, providing the molecular rational for H3K36me3 deposition to only occur co-transcriptionally.

**Fig. 5:**
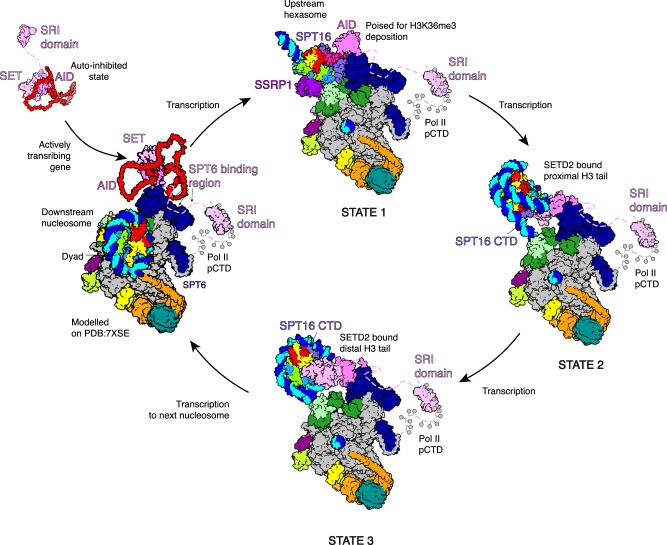
Model for co-transcriptional SPT6 mediated H3K36me3 deposition. SETD2 auto-inhibition is released by binding both the phosphorylated CTD of RPB1 and the SPT6 DLD of elongating Pol II. Pol II unwraps the downstream nucleosome during passage with SETD2 bound to SPT6, waiting for nucleosome transfer. FACT mediates nucleosome transfer upstream. The binding of the catalytic SET domain to the transferred hexasome is occluded by FACT and is in a poised state. Deposition of the final H2A–H2B dimer allows FACT dissociation from the nucleosome dyad and allows the SET domain to methylate the proximal H3 tail. The unwrapped nucleosome is stabilized by the SPT16 CTD. Further transcription rewraps the proximal histones. SETD2 repositions to the distal H3 tail whilst remaining in proximity to SPT6 DLD.

Our data converge with published results and lead to a three-step model for co-transcriptional H3K36me3 deposition. In the first step (Fig. 5), SETD2 binds to SPT6 through the SBR and positions the catalytic SET domain to the upstream edge of Pol II. In the second step, transcription of Pol II through the nucleosome goes along with FACT-mediated transfer of the incoming nucleosome from downstream to upstream DNA. This transfer positions a hexasome adjacent to the AID of SETD2 and reorientates the domain such that the auto-inhibition is overcome. Compared to a similar upstream state observed in yeast^28^, the FACT-bound hexasome forms additional contacts with the histone chaperone SPT6 and with a region of RTF1 we call the FACT “fastener” (Supplementary Fig. 18). The FACT “fastener” likely helps retain FACT-bound nucleosomes in proximity to Pol II during transcription, contributing unexpectedly to a previously suggested mechanism^28^. SPT6 extends the nucleosome cradle described previously^28^, indirectly acting as a histone chaperone. SETD2 is now poised for methylation, but FACT occludes the catalytic SET domain from binding. In a third step, further transcription then allows FACT to deposit the missing H2A–H2B dimer and to stabilize the partially unwrapped nucleosome, tethering to SPT6 limits the AID domain, allowing the catalytic SET domain to bind and methylate the proximal H3 tail (Fig. 5, State 2). Further transcription enables rewrapping of DNA around the proximal histones and repositioning of SETD2 to the distal H3 tail, facilitating methylation of the distal H3 tail and completing H3K36me3 deposition on the transcribed nucleosome (Fig. 5, State 3). This cycle continues throughout the gene body at each Pol II nucleosome passage event, thus resulting in H3K36me3 deposition in actively transcribed genes. Whilst this manuscript was in review, two additional studies were published that highlighted the importance of the SPT6-SETD2 interaction for mediating H3K36me3 on an upstream nucleosome in both yeast and metazoans^73,74^.

In summary, our results provide the molecular mechanism for co-transcriptional histone modification. Our mechanism resembles that of co-transcriptional RNA transcript modification during pre-mRNA capping and splicing, which also involves interactions with Pol II-associated general elongation factors^75–77^. We note that histone tail modifications other than H3K36me3 are also introduced co-transcriptionally^78^, including ubiquitylation of histone H2B at residue lysine-120 (H2BK120ub1), tri-methylation of histone H3 at residue lysine-4 (H3K4me3) and methylation of H3 at residue lysine-79 (H3K79me3). Some of the enzymes required for these modifications interact with the phosphorylated CTD or with Pol II elongation factors^18,20,79–81^. Whether these histone modifications are also introduced after nucleosome transfer to the wake of transcribing Pol II, and which interactions underlie their regulation, remains to be investigated.

## Methods

### Preparation of protein components

*Sus scrofa* RNA polymerase II was extracted from 0.5 – 2 kg pig thymus. *Homo sapiens* SPT6, PAF1c, RTF1, IWS1, SETD2, and FACT were expressed in *Trichoplusia ni* Hi5 cells and harvested after ~ 72 h (Expression Systems – CAT #94-002 F). V_0_ and V_1_ virus were produced in Sf9 (Oxford expression technology – CAT #600100) and Sf21 (Expression Systems – CAT #94-003 F) cells, respectively. *Homo sapiens* TFIIS, DSIF, and histones H2A, H2B, H3, H3K36M, and H4 were expressed for 3 h in BL21 (DE3) RIL *Escherichia coli* cells. *S. scrofa* Pol II and *H. sapiens* DSIF, TFIIS^7^, SPT6, PAF1c, RTF1^10,12^, FACT^34^ were purified, and human histones H2A, H2B, H3, H3K36M, and H4^82,83^ were purified as previously described.

*Homo sapiens* IWS1 was purified from 750 mL of Hi5 expression. All steps were performed at 4 °C. The cell pellet was resuspended and lysed by sonication in lysis buffer (300 mM NaCl, 20 mM Na·HEPES pH 7.4, 30 mM imidazole, 10% (v/v) glycerol, 1 mM DTT, 0.284 µg mL^–1^ leupeptin, 1.37 µg mL^–1^ pepstatin A, 0.17 mg mL^–1^ PMSF, and 0.33 mg mL^–1^ benzamidine). Cell debris were cleared by centrifugation, and the supernatant filtered through 0.8 µM syringe filter. The sample was applied to two pre-equilibrated 5 mL HisTrap HP column (Cytivia). The column was washed with 10 column volumes (CV) lysis buffer, 5 CV high salt buffer (1000 mM NaCl, 20 mM Na·HEPES pH 7.4, 30 mM imidazole, 10% (v/v) glycerol, 1 mM DTT, 0.284 µg mL^–1^ leupeptin, 1.37 µg mL^–1^ pepstatin A, 0.17 mg mL^–1^ PMSF, and 0.33 mg mL^–1^ benzamidine), and 5 CV low salt buffer (150 mM NaCl, 20 mM Na·HEPES pH 7.4, 30 mM imidazole, 10% (v/v) glycerol, 1 mM DTT, 0.284 µg mL^–1^ leupeptin, 1.37 µg mL^–1^ pepstatin A, 0.17 mg mL^–1^ PMSF, and 0.33 mg mL^–1^ benzamidine). A low salt equilibrated 5 mL HiTrap Q HP column (Cytivia) was attached to the base of the HisTrap column. The HisTrap column was eluted with a 3 CV long gradient in nickel elution buffer (150 mM NaCl, 20 mM Na·HEPES pH 7.4, 500 mM imidazole, 10% (v/v) glycerol, 1 mM DTT, 0.284 µg mL^–1^ leupeptin, 1.37 µg mL^–1^ pepstatin A, 0.17 mg mL^–1^ PMSF and 0.33 mg mL^–1^ benzamidine), before the column was removed. The remaining HiTrap Q column was washed with 5 CV low salt buffer before it was developed with a 9 CV high salt buffer gradient. Peak fractions were analyzed by denaturing polyacrylamide gel electrophoresis (SDS-PAGE) and coomassie staining. Fractions containing IWS1 were pooled and mixed with TEV protease, lambda protein phosphatase, and 1 mM MnCl_2_. The sample was dialyzed overnight using snake skin dialysis tubing (7 kDa molecular weight cutoff) (Thermo Fisher Scientific™) against 1 L lysis buffer containing 1 mM MnCl_2_. The sample was then applied to two pre-equlibrated 5 mL HisTrap HP column. The flow-through was collected and concentrated in a pre-equilibrated 50 kDa MWCO Amicon Ultra Centrifugal Filters (Milipore). The concentrated sample was then applied to a HiLoad 16/600 Superdex 200 pg column equilibrated in SEC buffer (300 mM NaCl, 20 mM Na·HEPES pH 7.4, 10% (v/v) glycerol, 1 mM DTT). Peak fractions were analyzed by SDS-PAGE and coomassie staining. Fractions containing pure IWS1 were pooled, concentrated in a pre-equilibrated 50 kDa MWCO Amicon Ultra Centrifugal Filters (Milipore), and snap frozen in liquid nitrogen. The protein was stored at − 70 °C until use.

SETD2 (residues 1446-2564) expressed in Hi5 insect cells as an 6xHis MBP fusion protein, and sonication and clarification were performed as described for IWS1. Clarified lysate was applied to an XK 16/20 column packed with amylose resin. The column was washed with 3 CV of lysis buffer (300 mM NaCl, 20 mM Na-HEPES, pH 7.4, 30 mM imidazole, 10% (v/v) glycerol, 1 mM DTT, 0.284 μg ml−1 leupeptin, 1.37 μg ml−l pepstatin A, 0.17 mg ml−1 PMSF and 0.33 mg ml−1 benzamidine) and SETD2 eluted with 2 CV of elution buffer (lysis buffer supplemented with 10 mM maltose). Eluted fractions were analyzed by SDS-PAGE, and fractions containing SETD2 were pooled and incubated with TEV protease for a minimum of 12 h. The cleaved sample was applied to two 5 mL HisTrap HP to remove the TEV protease and cleaved MBP tag. The columns were washed with one CV of lysis buffer, and the cleaved SETD2 recovered from the flow-through, concentrated in a 50 kDa MWCO Amicon Ultra Centrifugal Filter (Merck) and applied to HiLoad S200 16/600 pg column equilibrated with gel filtration buffer (300 mM NaCl, 20 mM Na-HEPES, pH 7.4, 10% (v/v) glycerol, 1 mM DTT). Peak fractions were assessed by SDS-PAGE, and fractions containing SETD2 were pooled and concentrated in a 50 kDa MWCO Amicon Ultra Centrifugal Filter (Merck), aliquoted, flash frozen in liquid nitrogen and stored at − 70 °C.

Protein truncations and deletions were expressed and purified in an identical fashion to the WT protein.

### Preparation of mononucleosomal and tetranucleosomal DNA constructs

DNA constructs for mononucleosomal templates were generated by a 50 mL PCR^6,7,36,82^. The template for amplification were vectors containing the Widom 601 nucleosome positioning sequence with a T-less cassette on the template strand to nucleosomal bp 32 or 64. PCR reactions were performed with two primers (forward primer: 5’-GCA GTC CAG TTA CGC TGG AGT C-3’; reverse primer: 5’-ATC AGA ATC CCG GTG CCG-3’). The PCR products were purified by anion exchange chromatography and digested with TspRI.

To generate nucleosome templates for cryo-EM studies that mimic transcription 159 or 174 bp into a nucleosome, a TspRI cleavage site placed either 69 or 84 bp downstream of the Widom 601 dyad. PCR reactions, purifications and TspRI digestions were performed as described above. Cleaved DNA products purified on 6% denaturing PrepCell PAGE (12% 19:1 acrylamide:bis-acrylamide, 8 M urea, 1x TBE running at 4 °C and 8 W in 0.5x TBE buffer) and subsequently concentrated by ethanol precipitation. Primers to generate the mismatch bubble (Non template 5’ – /5Phos/GCGGCCCTTGTGTTCAGGAGCCAGCAGGGAGCTGGGAGC, Template 5’ GCTCCCAGCTCCCTGCTGGCTCCGAGTGGGTTCTGCCGCGACAGTGAT) were mixed and annealed by slow cooling from 95 °C. Annealed primers were ligated to TspRI-cleaved Widom 601 sequences using T4 ligase as per the manufactures instructions.

DNA for tetranucleosomal arrays were generated from plasmid DNA, similar to a process described previously^84^. In brief, the plasmid DNA was extracted from XL1 Blue *E. coli* cells with GigaPrep extraction kits (Macherey-Nagel) and contained four Widom 601 sequences equally spaced by 30 bp linker DNA. The plasmid DNA was digested by EcoRV-HF at 37 °C for 10 h. The desired tetranucleosomal template DNA was purified by size-selective PEG precipitation at 800 mM NaCl and approx. 6-7% PEG-6000. The DNA was digested by TspRI at 65 °C for 10 h and purified on 6% denaturing PrepCell PAGE (6% 19:1 acrylamide:bis-acrylamide, 8 M urea, 1x TBE running at 4 °C and 8 W in 0.5x TBE buffer).

DNA for the mononucleosomal template used in the electromobility shift assays were generated by 50 mL PCR^6,7,36,82^. The template for amplification was vector containing the Widom 601 nucleosome. PCR reactions were performed with two primers (forward primer: 5’-ATC GAT GTA TAT ATC TGA CAC GTG CCT G-3’; reverse primer: 5’-ATC AGA ATC CCG GTG CCG AGG C-3’).

### Nucleosome reconstitutions

All reconstitution were performed with WT or H3K36M containing octamers using the salt-gradient dialysis method^83^. The ratio between DNA and histone octamer for successful reconstitution of mononucleosome or tetranucleosomes were determined by a titration series of histone octamer to DNA. For tetranucleosomes, the reconstitution titration was analyzed by BanI digestion and agarose PAGE. The minimal histone octamer concentration for complete inhibition of BanI cleavage was chosen to generate tetranucleosomal constructs. Nucleosomes used for cryo-EM studies were reconstituted with histone octamers containing an H3K36M mutation. The concentration of final nucleosome substrates were calculated by the extinction coefficient and the absorption at 280 nm.

### RNA extension assays with methylation

RNA extension assays were performed on mononucleosomal and tetranucleosomal constructs similarly as described^6,7,34^.

Mononucleosome extension assay in Fig. 1a were performed in a final volume of 18 µL. RNA (150 nM) containing a 5’ Cy5 label and nucleosomal template (60 nM) were mixed and incubated for 10 min on ice. *S. scrofa* Pol II (150 nM) was added to the reaction and incubated for 10 min on ice. DSIF (225 nM), SPT6 (225 nM), RTF1 (225 nM), PAF1c (225 nM), P-TEFb (250 nM), IWS1 (0.450 nM), FACT (300 nM), 3’d ATP (0.5 mM), SETD2 (6 µM), and water were added to the reaction. Compensation buffer then added such that the final buffer composition in the reaction to 90 mM NaCl, 20 mM Na·HEPES pH 7.4, 4 mM MgCl_2_, 4% (v/v) glycerol, and 1 mM DTT, and 10 µM ZnCl_2_. The reactions were incubated for 15 min at 30 °C. Transcription was started with the addition of TFIIS (90 nM), CTP, GTP, UTP, ATP (300 µM each) and S-Adenosyl methionine (SAM) (10 µM). Nucleotides or ATP were omitted from reactions without transcription or to stall the Pol II at the end of the T-less cassette. Reactions were allowed to proceed for 10 min at 30 °C. To monitor RNA extension, five microliters of the reactions were taken and quenched with 2x stop buffer (6.4 M urea, 50 mM EDTA, pH 8.0, 2x TBE buffer). Proteinase K (40 µg) (New England Biolabs) and incubated for 30 min at 37 °C. Samples were denatured at 95 °C for 10 min and separated by denaturing PAGE (12% acrylamide:bis-acrylamide 19:1, 8 M urea, 1x TBE buffer, ran at 300 V in 0.5x TBE buffer for approx. 40 min). To monitor H3K36me3, 12 µL of the reaction was mixed with 6 µL 4x LDS dye (Thermo Fisher Scientific). Samples were denatured for 2 min at 95 °C, and 12 µL were subjected to denaturing PAGE and subsequently transferred onto nitrocellulose membranes (GE Healthcare Life Sciences). To probe for H3K36me3, the membrane was blocked with milk before incubating with primary antibody overnight at 4 °C in a 1/10,000 dilution (H3K36me3-mouse - Active Motif, REF: 61021 / AB_2614986), followed by washing with PBST. An HRP conjugated secondary antibody (Goat anti-Mouse IgG HRP - ID:AB_228307, Cat #31430) was applied at a dilution of in 1/20,000 for 1 hour at room temperature before additional washes with 1 x PBST. Enhanced chemifluorescent HRP substrate (Thermo Fisher Scientific) was added prior to imaging.

Tetranucleosome extension assays in Fig. 3c were performed in a final volume of 15 µL. RNA (150 nM) containing a 5’ Cy5 label and nucleosomal template (60 nM) were mixed and incubated for 10 min on ice. *S. scrofa* Pol II (150 nM) was added to the reaction and incubated for 10 min on ice. DSIF (225 nM), SPT6 (225 nM), RTF1 (225 nM), PAF1c (225 nM), P-TEFb (250 nM), IWS1 (0.450 nM), FACT (300 nM), ATP (1 mM), SETD2 (0.6 µM), and water were added to the reaction. Compensation buffer was then added such that the final buffer composition in the reaction was 90 mM NaCl, 20 mM Na·HEPES pH 7.4, 4 mM MgCl_2_, 4% (v/v) glycerol, 1 mM DTT, and 10 µM ZnCl_2._ The reactions were incubated for 15 min at 30 °C. Transcription was started with the addition of TFIIS (90 nM), CTP, GTP, UTP (300 µM each), and SAM (4 µM. Reactions were allowed to proceed for 30 min at 30 °C. Samples from tetranulceosomal reactions were treated with DNaseI due to the longer DNA and RNA. Five microliters from tetranucleosomal reactions were mixed with 0.6 µL 10x CaCl_2_ buffer (100 mM Na·HEPES pH 7.4, 50 mM CaCl_2_, 25 mM MgCl_2_) and proteinase K (40 µg). The samples were incubated for 20 min at 55 °C, cooled down to 37 °C, before 1.5 U DNaseI (RNase-free, Thermo Scientific™) were added. The samples were incubated at 37 °C for 20 min, then quenched with 2x stop buffer, denatured for 10 min at 95 °C and separated by 6% denaturing PAGE gels (6% acrylamide:bis-acrylamide 19:1, 8 M urea, 1x TBE buffer, ran at 300 V in 0.5x TBE buffer for approx. 30 min). The 5′ Cy5 label of the extended RNA product was visualized on a Typhoon FLA 900 biomolecular imager using an excitation wavelength of 635 nm. To detect the RNA ladder, gels were stained with SYBR™ Gold and visualized at an excitation wavelength of 473 nm. Raw scans available as Source Data file.

### Filter binding assays

For quantification of in vitro methylation of the tetranucleosome extension assays, experiments were performed as outlined above, substituting cold SAM with 4 μM 3H SAM (Perkin Elmer). 10 μL of the reaction were spotted on nitrate cellulose membranes and washed with 3 mL of 20 mM HEPES (pH 7.4) under vacuum. Membranes were dissolved and methylated products quantified by liquid scintillation. Data were normalized to the average (*N* = 3) values for the presence or absence of SETD2. Reactions were performed in triplicate. Data displayed as box plots, each point reflects one replicate (*N* = 3), depicted as mean ± s.d. Unnormalized data available as Source Data file.

### Sample preparation for cryo-EM analysis

#### State 1 complex

The methylation-competent elongation complex with an upstream transferred hexasome was assembled in a final buffer containing 70 mM NaCl, 20 mM Na·HEPES pH 7.4, 3 mM MgCl_2_, 1 mM DTT, and 4% (v/v) glycerol. The reaction volume was 300 µL. Complex formation was performed similarly to the RNA extension assays. 5’ Cy5 labeled RNA (960 nM) and nucleosomal template (480 nM) were mixed and incubated on ice for 10 min. *S. scrofa* Pol II (400 nM) was added and the reaction was incubated on ice for a further 10 min. DSIF (800 nM), SPT6 (800 nM), PAF1c (800 nM), RTF1 (800 nM), P-TEFb (400 nM), AKT3 (400 nM), IWS1 (2.4 µM), SETD2 (1.6 µM), 3’dATP (0.5 mM) (Jena Bioscience), FACT (800 nM), and water was added. Compensation buffer was then added to bring the reaction final conditions to those listed above, and the reaction was then incubated for 15 min at 30 °C. Transcription elongation was started with the addition of TFIIS (240 nM) and CTP (0.1 mM), GTP (0.1 mM) and UTP (0.1 mM), and was allowed to continue for 30 min at 30 °C to the end of the T-less cassette of the template strand at nucleosomal bp 64. The reaction was applied onto a 2 mL glycerol grafix gradient containing 10-30% (v/v) glycerol, 65 mM NaCl, 20 mM Na·HEPES pH 7.4, 3 mM MgCl_2_, 1 mM DTT, and 0.0175% (v/v) glutaraldhyde in the heavy solution. The gradient was spun in a TLS-55 swinging rotor (Beckman Colter) for 3 h at 55,000 x *g* and 4 °C. After the centrifugation, the gradient was fractionated in 100 µL steps from the top and quenched with 8 mM aspartate and 10 mM lysine for 10 min on ice. Fractions were analyzed using 12% denaturing PAGE (as described in RNA extension) and 3–12% nativePAGE™ (Invitrogen™). Fractions containing the complex (Supplementary Fig. 1) dialyzed for 3 h in 50 mM NaCl, 20 mM Na·HEPES, pH 7.4, 20 mM Tris-Cl, pH 7.5, and 1 mM DTT.

For grid preparation, R2/2 UltrAuFoil grids (Quantifoil) were glow-discharged for 100 s. DDM was added to the dialyzed sample to a final concentration of 0.0025%. 3 µL sample was applied to both sides of the grids and incubated for ~ 5 s at 4 °C and 100% humidity. The grids were blotted with a blot force of 5 for 3 s before plunging into liquid ethane using a Vitrobot Mark IV (Thermo Fisher). Grids were clipped and stored in liquid nitrogen until cryo-EM analysis.

#### State 2 complex

The methylation-competent elongation complex with SETD2 bound to the proximal H3 tail of an upstream nucleosome was assembled in a final buffer containing 80 mM NaCl, 20 mM Na·HEPES pH 7.4, 3 mM MgCl_2_, 1 mM DTT, and 4% (v/v) glycerol. The reaction volume was 90 µL. 5’ FAM labeled RNA (1.67 µM)(/56-FAM/rUrUrArArGrGrArArUrUrArArGrUrCrGrUrGrCrGrUrCrUrArArUrArArCrCrGrGrArGrArGrGrGrArArCrCrCrArCrU) and the ligated ( + 159 bp) nucleosomal template (0.86 µM) were mixed with SETD2 (8.6 µM) and SAM (0.1 mM) on ice for 10 min. *S. scrofa* Pol II (1 µM) was added and the reaction was incubated for a further 10 min on ice. DSIF (1.5 µM), SPT6 (1.5 µM), IWS1 (1.5 µM), RTF1 (2 µM), PAF1c (1.5 µM), P-TEFb (0.4 µM), ATP (0.5 mM), and water were added. Compensation buffer was then added to bring the final reaction conditions to those listed above, and the sample was then incubated at 30 °C for 15 min. After centrifugation, the complex was purified by gel filtration using a Superose 6 Increase (3.2/300) pre-equilibrated with 50 mM NaCl, 20 mM Na·HEPES pH 7.4, 3 mM MgCl_2_, 1 mM DTT, and 4% glycerol, in the presence of FACT (Supplementary Fig. 11a). A single peak fraction containing all components was crosslinked with 0.1% w/v glutaraldehyde on ice for 10 min before quenching with 16 mM aspartate and 4 mM lysine.

For grid preparation, R2/2 UltrAuFoil grids (Quantifoil) were glow-discharged for 100 s. 2.5 µL sample was applied to both sides of the grids and incubated for ~15 s at 4 °C and 100% humidity. The grids were blotted with a blot force of 5 for 3 s before plunging into liquid ethan using a Vitrobot Mark IV (Thermo Fisher).

#### State 3 complex

The methylation-competent elongation complex with SETD2 bound to the distal H3 tail of an upstream nucleosome was assembled on ligated a nucleosomal template (+ 174 bp), and the complex purified and frozen in an identical manner to the proximal H3 bound complex (Supplementary Fig. 11b).

### Cryo-EM analysis and image processing

For State 1, cryo-EM data were collected under near-identical conditions on two separate grids during different collection periods. Data was acquired at a nominal magnification of 81,000 ×, corresponding to a calibrated pixel size of 1.05 Å/pixel, using a K3 direct electron detector (Gatan) on a Titan Krios transmission electron microscope (Thermo Fisher Scientific) operated at 300 kV. Images were collected in EFTEM mode using a Quantum LS energy filter with a slit width of 20 eV. Images were collected in electron counting mode with an applied defocus range of − 0.5 to − 2.0 μm. The SerialEM^85^ software was used for automated data acquisition. All pre-processing of collected movies (motion correction, dose weighting, CTF estimation and particle picking) were performed using Warp^86^.

For dataset 1, we collected 45,705 micrographs with a dose rate of 14.45 e^−^/pixel/s for 3.05 s, resulting in a total dose of 39.98 e^−^/Å^2^ that was fractionated into 40 movie frames. Micrographs with bad CTF fits in Warp were excluded from further processing. We extracted 5,835,867 picked particles with a box size of 512 pixels and binned 5x to a pixel size of 5.25 Å/pixel using RELION 3.1^87^. These particles were subjected to interactive rounds of 2D classification and heterogeneous refinement in CryoSPARC^88^ using initial models generated by ab initio reconstruction. Classes with good particles for Pol II were re-extracted in RELION^87^ (binned 2 x, pixel size 2.1 Å/pixel) and further cleaned using 2D and 3D classification. Finally, particles were re-extracted without binning and focused refinement, with a mask around Pol II was performed. These particles (432,698) were subjected to iterative rounds of CTF refinement and Bayesian polishing, resulting in a 3.62 Å reconstruction, encompassing the activated elongation complex and additional upstream density. For dataset 2, we collected 55,723 micrographs with a dose rate of 15.83 e^−^/pixel/s for 2.82 s, resulting in a total dose of 40.49 e^−^/Å2 that was fractionated into 40 movie frames. Micrograph curation, pre-processing and initial particle cleanup was performed as described in dataset1. After CTF refinement and polishing, 804,464 particles contributed to a 2.92 Å reconstruction and was merged with dataset 1. Polishing artifacts were present in both datasets were filtered based on X, Y coordinate on the micrographs.

To determine the composition of the upstream density, we applied a generous soft mask around the upstream half of Pol II and subtracted the signal outside of this region using RELION 3.1^68,87,89^. Subsequent 3D classification and refinement identified a subset of particles that produced of 3.89 Å resolution reconstruction (MAP 1) from which we could observe a FACT- bound hexasome, immediately upstream of the Pol II. To improve the resolution, we repeated the signal subtraction, using a tighter mask around the FACT-hexasome complex, and repeated 3D classification and refinement. The resulting reconstruction (MAP 2) yielded improved local resolution and an overall reconstruction at 3.86 Å resolution. To obtain an overall reconstruction of Pol II and the FACT-hexasome complex, particles from MAP 2 (after reversion of the signal subtraction) were back-projected using the angles obtained from the refinement of MAP 1 in RELION^87^. This produced the best consensus map of both the polymerase and FACT-hexasome complex at 6.4 Å resolution. To resolve the flexibility in SPT6, the signal was subtracted outside a soft mask around SPT6. Subsequent 3D classification and refinement identified a two subset of particles, one containing canonical SPT6^10^ and a second subset in which SPT6 rotated upwards (MAP 4). Reversion of the signal subtraction of both subsets and subsequent global refinement resulted in one map with a stable FACT-bound hexasome (for the rotated SPT6 subset) and the second map without density for FACT or a hexasome. ~40% of particles from MAP 4 overlap with MAP 2, whilst only ~2.5% of particles from the canonical SPT6 position overlap with MAP 4.

For State 2, cryo-EM data were collected under near-identical conditions to above, including nominal magnification, pixel size, detector, energy filter and defocus range. All pre-processing of the collected movies (motion correction, dose weighting, CTF estimation and particle picking) were performed using Warp^86^. We collected 77,138 micrographs with a dose rate of 16.39 e^−^/pixel/s for 2.43 s, resulting in a total dose of 39.83 e^−^/Å^2^ that was fractionated into 40 movie frames. Micrographs with bad CTF fits in Warp were excluded from further processing. We extracted 4,613,946 picked particles with a box size of 512 pixels and binned 4 x to a pixel size of 4.2 Å/pixel using RELION 3.1^68,87,89^. These particles were subjected to interactive rounds of 2D classification and heterogeneous refinement in CryoSPARC^88^ using initial models generated by ab initio reconstruction. Classes with good particles for Pol II re-extracted in RELION (binned 2x, pixel size 2.1 Å/pixel) and further cleaned using 2D and 3D classification and decreasing spherical masks around Pol II. Finally, particles were re-extracted without binning and focused refinement performed. These particles (~750,00) were subjected to iterative rounds of CTF refinement and Bayesian polishing, resulting in a 2.63 Å reconstruction, encompassing the activated and additional upstream density.

To determine the composition of the upstream density, we applied a generous soft mask around the upstream density and subtracted the signal outside of this region using RELION 3.1^68,87,89^. Due to severe heterogeneity, initial angular assignments required 2D classification (performed in CryoSPARC) and addition rounds of heterogeneous refinement. Well, aligning particles of SETD2 bound to the nucleosome were imported back into RELION 3.1^68,89^ for additional focused classification (Supplementary Fig. 10c) to remove ~20,000 particles that contained two SETD2 molecules bound. Remaining particles resulted in an overall reconstruction of SETD2 bound to the nucleosome at 4.25 Å from approximately 80,000 particles (MAP 1). These particles were selected from the consensus refinement stack, thereby recovering angles Pol II refinement angles. Multibody refinement performed in RELION 3.1^68^ was used to determine the principal components of nucleosome motion, relative to Pol II. Given the unimodal distribution, a subset of particles was selected based on the eigenvalue for component 1 ( +/− 10) and re-extracted to center the particles between Pol II and the nucleosome. Final refinement of the centered particles was performed using a spherical mask of 250 Å diameter (MAP 3). For the classification of SPT6, an initial soft mask around SPT6 was used for subtraction. Focused 3D classification with local angular sampling was used to select a subset of well-aligning particles. Refinement steps that utilized Blush in RELION 5^90^ are indicated in Supplementary Fig. 10c. A second focused classification, without image shift alignments, was performed using a tight spherical mask around the DLD of SPT6. Subsequent refinement of particles containing SETD2 resulted in a 3.82 Å reconstruction (MAP 2). 18.5% of particles in MAP 1 overlap with MAP 2, suggesting a subset of the SETD2 particles remain tethered to SPT6 whilst simultaneously binding the nucleosome.

For State 3, cryo-EM data was collected in an identical fashion to State 2. All pre-processing of the collected movies (motion correction, dose weighting, CTF estimation and particle picking) were performed using Warp^86^. We collected 74,840 micrographs with a dose rate of 16.40 e^−^/pixel/s for 2.44 s, resulting in a total dose of 40.01 e^−^/Å^2^ that was fractionated into 40 movie frames. Micrographs with bad CTF fits in Warp were excluded from further processing. We extracted 9,589,557 picked particles with a box size of 512 pixels and binned 4x to a pixel size of 4.2 Å/pixel using RELION 3.1^68,87,89^. Data processing was carried out in a similar fashion to State 2 and as outlined in Supplementary Fig. 14.

### Model building and refinement

To build a model for State 1 into MAP 1-4, Alphafold2 models of human SSRP1 and SPT16 were placed within the cryo-EM density using ISOLDE flexible fitting^91^. To guild fitting, PDB 7XTI^28^ was used as a reference to restrain the Alphafold2 models. The nucleosome (PDB 2CV5)^92^ was rigid body docked into the density, using ChimeraX^93,94^ and the proximal H2A–H2B dimer and DNA removed. Due to the anisotropic nature of the density, the sequence register of the RNA polymerase II active site (in MAP 3) could not be determined. Therefore, the DNA was modeled based on the designed stall site located 64 bp into the nucleosome. ISOLDE^91^ was used to fit the parts of SPT4 and SPT6, present in the reconstruction, from their respective models in 6TED^10^. To model the FACT “fastener”, we used ColabFold AlphaFold2 w/ MMseqs2^59^ to generate a model of the SPT16 (residues 457–930) and RTF (residues 266–315) that was subsequently used as a guide to place the FACT “fastener” into the observed density. Similarly, ColabFold^59^ was used to model a hexasome bound by SPT16 that assisted in the modeling of the SPT16 CTD. For building Pol II, 6TED^10^ was positioned into MAP 3, and protein chains outside of the map density were flexibly fitted using ISOLDE^91^ or removed if density was absent. MAP 4 was used to correctly position SPT6 and build the SPT6-SETD2 interface guided by the ColabFold prediction. The Alphafold2 model of the SETD2 AID was rigid-body docked into MAP 1, and flexible regions removed. Reference model restraints were applied to prevent secondary structure deterioration of the model in regions of lower local resolution

To model the nucleosome in State 2, PDB 7EA8^58^ was rigid-body fitted into State 2 MAP 1. Histone residues where mutated to match the sequences used. For SPT6, the Alphafold2 model was fitted to PDB 6TED^10^ and adjusted with ISOLDE. Residues outside the available cryo-EM density for State 2 MAP 2 were removed. The complete model of State 2 was built by fitting PDB 6TED^10^ and the models for State 2 MAP 1 and 2 into MAP 3 and B-DNA modeled between the active site of Pol II and the unwrapped nucleosome using COOT^95^. Given the flexible nature, the AID was not modeled in State 2. The CTD of SPT16 was model based on PDB 8I17^67^. For figures, the AlphaFold2 model of the AID was rigid-body docked into MAP 1 cryo-EM density in ChimeraX^93,94^. A similar approach was taken to model State 3.

All models were refined using the Phenix.real_space_refine tool in the PHENIX package, we reference model restraints^96,97^.

### Crosslinking mass spectrometry

For crosslinking mass spectrometry, the activated elongation complex transcribed to nucleosomal base pair 64 with an upstream FACT-bound hexasome was assembled similarly to the formation for cryo-EM. The differences of the complex assembly compared to the cryo-EM sample preparation are listed below. The final volume of the assembly was 600 µL with protein and nucleosome concentrations unchanged. To stall the polymerase at the end of the T-less cassette, 1 mM 3’dATP (Jena Bioscience) was used. The complex assembly was applied to a 4 mL glycerol gradient containing 15–45% (v/v) glycerol, 65 mM NaCl, 20 mM Na·HEPES pH 7.4, 3 mM MgCl_2_, and 1 mM DTT. The gradient was spun in a SW55 Ti swinging rotor (Beckman Colter) for 16 h at 55,000 rpm and 4 °C. Post centrifugation, the gradient was fractionated in 200 µL steps from the top. Fractions containing the assembled complex were pooled and crosslinked with 2 mM bis(sulfosuccinimidyl)suberate (BS3) on ice before being quenched by 100 mM Tris-HCl, pH 7.4. The complexes were pelleted by ultracentrifugation in an S150AT rotor (Thermo Fisher Scientific). The pellet was solubilized in 50 mM ammonium bicarbonate (pH 8.0) supplemented with 4 M urea, reduced with 5 mM DTT and alkylated with 17 mM iodoacetamide. The sample was diluted with 50 mM ammonium bicarbonate to reduce urea concentration to 1 M and digested with trypsin (Promega) in a 1:20 enzyme-to-protein ratio (w/w) at 37 °C overnight. Peptides were reverse-phase extracted using SepPak Vac tC18 1cc/50 mg (Waters) and eluted with 50% acetonitrile (ACN) / 0.1% trifluoroacetic acid (TFA). The eluate was dried in a vacuum concentrator (Eppendorf). Dried peptides derived from 33 pmol of the complex were dissolved in 35 µl of 2% ACN / 20 mM ammonium hydroxide and separated by reverse phase HPLC at basic pH using an xBridge C18 3.5 µm 1 x 150 mm column (Waters) at a flow rate of 60 µl/min at 24 °C. Buffers A and B for mobile phase were 20 mM ammonium hydroxide, pH 10, and 80% ACN / 20 mM ammonium hydroxide, pH 10, respectively. Peptides were bound to a column pre-equilibrated with 5% buffer B and eluted over 64 min using the following gradient: 5%B (min 0–4), 5-8%B (min 5–7), 8-36%B (min 8–41), 36–45%B (min 42–49), 45–95%B (min 50), 95%B (min 51–55), 95-5%B (min 56-57), 5%B (min 58–63). Fractions of 60 µl were collected. Peptides eluted between minutes 3 and 55 were vacuum dried and dissolved in 5% ACN / 0.1% TFA for subsequent uHPLC-ESI-MS/MS analysis. The fractions were injected into a Dionex UltiMate 3000 uHPLC system (Thermo Fisher Scientific) coupled to a Thermo Orbitrap Exploris mass spectrometer and measured twice with a 60 and twice with a 90 min method. For uHPLC, a C18 PepMAP 100 trap column (0.3 × 5 mm, 5 μm, Thermo Fisher Scientific) and a custom 30 cm C18 main column (75 µm inner diameter packed with ReproSil-Pur 120 C18-AQ beads, 3 µm pore size, Dr. Maisch GmbH) was used. Mobile phase was formed using buffers A (0.1% formic acid) and B (80% ACN / 0.08% formic acid). Peptide separation was achieved by applying a linear gradient of 11–45%B (min 3 to 42 in a 60 min method or min 3 to 72 in a 90 min method), followed by 45–52%B (min 43–47 in a 60 min method or min 73–78 in a 90 min method). MS settings were as follows: MS1 resolution, 120000; MS1 scan range, 350–1550 m/z; MS1 normalized AGC target, 300%; MS1 maximum injection time, auto; cycle time (Top Speed), 3 s; intensity threshold, 1E4; MS2 resolution, 60000; isolation window, 1.6 Th; normalized collision energy, 30%; MS2 AGC target, 75%; MS2 maximum injection time, 128 ms. Only precursors with a charge state of 3–8 were selected for MS2 using a dynamic exclusion of 25 s. Protein-protein crosslinks were identified by searching Thermo raw files against a custom database of 31 proteins using pLink2.3.11 software (https://pfind.ict.ac.cn/se/plink/and filtered at a false discovery rate (FDR) of 5% according to the recommendations of the developer^98,99^. Filtered crosslinks are reported in Supplementary Data 1.

### Electromobility shift assay

For electromobility shift assays, truncated SETD2 constructs (between 0 and 5 µM) were incubated with mononucleosomes (0.2 µM) at 4 °C for 1 h in a final buffer containing 20 mM Na·HEPES pH 7.4, 2 mM MgCl_2_, 50 mM NaCl and 0.2 mg/mL BSA. 1 µL of sample was mix with 1 µL of loading dye (50% (v/v) glycerol with OrangeG) and separated for 1 h at 4 °C on a 5% native 37.5:1 (acrylamide/bis-acrylamide) PAGE. PAGE gel was stained with SYBR^TM^ Gold (Invitrogen).

### SETD2 auto-inhibitory domain binding assays

SET and AID domains (2 µM) were incubated together in equimolar concentrations in a final buffer containing 20 mM Na·HEPES pH 7.4, 150 mM NaCl (or 100 mM as indicated in Supplementary Fig. 17d) and 4% (v/v) glycerol at 4 °C for 30 min. The sample was applied to a Superose 6 Increase 3.2/300 column (Cytiva), pre-equilibrated in incubation buffer. Relevant fractions were analyzed by SDS-PAGE and coomassie staining.

### Figure generation

Figures were generated using UCSF ChimeraX (version 1.6)^93,94^.

### Quantification and statistical analysis

Quantification of signal from denaturing PAGE gels were performed using the integrated density function of ImageJ2 2.3.0. No statistical analysis were performed.

### Material availability

Materials are available from the corresponding author, Patrick Cramer, upon request under a material transfer agreement with the Max Planck Society.

## Supplementary information

Supplementary Information

Description of additional supplementary files

Supplementary Dataset 1

Transparent Peer Review file

## Source data

Source data

## Data Availability

The cryo-EM reconstructions and final model for State 1, have been deposited in the Electron Microscopy Data Base (EMDB) under ID codes EMD-54538, EMD-54541, EMD-54537, and EMD-54542 for maps 1–4, respectively, and in the Protein Data Bank (PDB) under ID code 9S3G. For State 2 maps 1–3, EMDB codes EMD-51643, EMD-54247, and EMD-54401 and PDB 9GW2, 9RTN, and 9RZE. For State 3 maps 1–3. EMDB codes EMD-54399, EMD-54400, EMD-54425 and PDB 9RZC, 9RZD, and 9S0U. Initial models used for reference restraints or modeling building. 7XTI. 2CV5. 6TED. 7EA8. 8I17. CXL-MS data are available via ProteomeXchange with identifier PXD067197. Source data are provided in this paper.

## References

[1] Venkatesh, S. & Workman, J. L. Histone exchange, chromatin structure and the regulation of transcription. *Nat. Rev. Mol. Cell Biol.* **16**, 178–189 (2015). 25650798 10.1038/nrm3941

[2] Petesch, S. J. & Lis, J. T. Overcoming the nucleosome barrier during transcript elongation. *Trends Genet.* **28**, 285–294 (2012). 22465610 10.1016/j.tig.2012.02.005 PMC3466053

[3] Teves, S. S., Weber, C. M. & Henikoff, S. Transcribing through the nucleosome. *Trends Biochem. Sci.* **39**, 577–586 (2014). 25455758 10.1016/j.tibs.2014.10.004

[4] Kulaeva, O. I., Hsieh, F.-K., Chang, H.-W., Luse, D. S. & Studitsky, V. M. Mechanism of transcription through a nucleosome by RNA polymerase II. *Biochim. Biophys. Acta* **1829**, 76–83 (2013). 22982194 10.1016/j.bbagrm.2012.08.015 PMC3535581

[5] Studitsky, V. M., Nizovtseva, E. V., Shaytan, A. K. & Luse, D. S. Nucleosomal barrier to transcription: Structural determinants and changes in chromatin structure. *Biochem. Mol. Biol. J.* **2**, 8 (2016). 27754494 10.21767/2471-8084.100017 PMC5041593

[6] Farnung, L., Ochmann, M., Engeholm, M. & Cramer, P. Structural basis of nucleosome transcription mediated by Chd1 and FACT. *Nat. Struct. Mol. Biol.* **28**, 382–387 (2021). 33846633 10.1038/s41594-021-00578-6 PMC8046669

[7] Farnung, L., Ochmann, M., Garg, G., Vos, S. M. & Cramer, P. Structure of a backtracked hexasomal intermediate of nucleosome transcription. *Mol. Cell* **82**, 3126–3134 (2022). 35858621 10.1016/j.molcel.2022.06.027

[8] Orphanides, G., LeRoy, G., Chang, C.-H., Luse, D. S. & Reinberg, D. FACT, a Factor that facilitates transcript elongation through nucleosomes. *Cell* **92**, 105–116 (1998). 9489704 10.1016/s0092-8674(00)80903-4

[9] Orphanides, G. & Reinberg, D. RNA polymerase II elongation through chromatin. *Nature* **407**, 471–476 (2000). 11028991 10.1038/35035000

[10] Vos, S. M., Farnung, L., Linden, A., Urlaub, H. & Cramer, P. Structure of complete Pol II–DSIF–PAF–SPT6 transcription complex reveals RTF1 allosteric activation. *Nat. Struct. Mol. Biol.* **27**, 668–677 (2020). 32541898 10.1038/s41594-020-0437-1

[11] Mason, P. B. & Struhl, K. Distinction and relationship between elongation rate and processivity of RNA polymerase II in vivo. *Mol. Cell* **17**, 831–840 (2005). 15780939 10.1016/j.molcel.2005.02.017

[12] Vos, S. M. et al. Structure of activated transcription complex Pol II–DSIF–PAF–SPT6. *Nature* **560**, 607–612 (2018). 30135578 10.1038/s41586-018-0440-4

[13] Žumer, K. et al. Two distinct mechanisms of RNA polymerase II elongation stimulation in vivo. *Mol. Cell* **81**, 3096–3109 (2021). 34146481 10.1016/j.molcel.2021.05.028

[14] Warner, J. L., Lux, V., Veverka, V. & Winston, F. The histone chaperone Spt6 controls chromatin structure through its conserved N-terminal domain. *Mol. Cell* **85**, 3407–3424 (2025). 10.1016/j.molcel.2025.08.020 PMC12453606 40972526

[15] Cao, Q.-F. et al. Characterization of the human transcription elongation factor Rtf1: evidence for vonoverlapping functions of Rtf1 and the Paf1 complex. *Mol. Cell. Biol.* **35**, 3459–3470 (2015). 26217014 10.1128/MCB.00601-15 PMC4573716

[16] Simic, R. et al. Chromatin remodeling protein Chd1 interacts with transcription elongation factors and localizes to transcribed genes. *EMBO J.* **22**, 1846–1856 (2003). 12682017 10.1093/emboj/cdg179 PMC154471

[17] Warner, M. H., Roinick, K. L. & Arndt, K. M. Rtf1 is a multifunctional component of the Paf1 complex that regulates gene expression by directing cotranscriptional histone modification. *Mol. Cell. Biol.* **27**, 6103–6115 (2007). 17576814 10.1128/MCB.00772-07 PMC1952162

[18] Fetian, T. et al. Paf1 complex subunit Rtf1 stimulates H2B ubiquitylation by interacting with the highly conserved N-terminal helix of Rad6. *Proc. Natl. Acad. Sci. USA* **120**, e2220041120 (2023). 37216505 10.1073/pnas.2220041120 PMC10235976

[19] Ng, H. H., Dole, S. & Struhl, K. The Rtf1 component of the Paf1 transcriptional elongation complex is required for ubiquitination of histone H2B. *J. Biol. Chem.* **278**, 33625–33628 (2003). 12876293 10.1074/jbc.C300270200

[20] Piro, A. S., Mayekar, M. K., Warner, M. H., Davis, C. P. & Arndt, K. M. Small region of Rtf1 protein can substitute for complete Paf1 complex in facilitating global histone H2B ubiquitylation in yeast. *Proc. Natl. Acad. Sci. USA* **109**, 10837–10842 (2012). 22699496 10.1073/pnas.1116994109 PMC3390850

[21] Van Oss, S. B. et al. The histone modification domain of Paf1 complex subunit Rtf1 directly stimulates H2B ubiquitylation through an interaction with Rad6. *Mol. Cell* **64**, 815–825 (2016). 27840029 10.1016/j.molcel.2016.10.008 PMC5131541

[22] Wood, A., Schneider, J., Dover, J., Johnston, M. & Shilatifard, A. The Paf1 complex is essential for histone monoubiquitination by the Rad6-Bre1 complex, which signals for histone methylation by COMPASS and Dot1p. *J. Biol. Chem.* **278**, 34739–34742 (2003). 12876294 10.1074/jbc.C300269200

[23] Orphanides, G., Wu, W.-H., Lane, W. S., Hampsey, M. & Reinberg, D. The chromatin-specific transcription elongation factor FACT comprises human SPT16 and SSRP1 proteins. *Nature* **400**, 284–288 (1999). 10421373 10.1038/22350

[24] Belotserkovskaya, R. FACT Facilitates transcription-dependent nucleosome alteration. *Science* **301**, 1090–1093 (2003). 12934006 10.1126/science.1085703

[25] Hsieh, F.-K. et al. Histone chaperone FACT action during transcription through chromatin by RNA polymerase II. *Proc. Natl. Acad. Sci. USA* **110**, 7654–7659 (2013). 23610384 10.1073/pnas.1222198110 PMC3651417

[26] Pavri, R. et al. Histone H2B monoubiquitination functions cooperatively with FACT to regulate elongation by RNA polymerase II. *Cell* **125**, 703–717 (2006). 16713563 10.1016/j.cell.2006.04.029

[27] Wang, T. et al. The histone chaperone FACT modulates nucleosome structure by tethering its components. *Life Sci. Alliance* **1**, e201800107 (2018). 30456370 10.26508/lsa.201800107 PMC6238592

[28] Ehara, H., Kujirai, T., Shirouzu, M., Kurumizaka, H. & Sekine, S. Structural basis of nucleosome disassembly and reassembly by RNAPII elongation complex with FACT. *Science* **377**, eabp9466 (2022). 35981082 10.1126/science.abp9466

[29] Jamai, A., Puglisi, A. & Strubin, M. Histone chaperone Spt16 promotes redeposition of the original H3-H4 histones evicted by elongating RNA polymerase. *Mol. Cell* **35**, 377–383 (2009). 19683500 10.1016/j.molcel.2009.07.001

[30] Jeronimo, C., Poitras, C. & Robert, F. Histone recycling by FACT and Spt6 during transcription prevents the scrambling of histone modifications. *Cell Rep.* **28**, 1206–1218 (2019). 31365865 10.1016/j.celrep.2019.06.097

[31] Jeronimo, C., Watanabe, S., Kaplan, C. D., Peterson, C. L. & Robert, F. The histone chaperones FACT and Spt6 restrict H2A.Z from intragenic locations. *Mol. Cell* **58**, 1113–1123 (2015). 25959393 10.1016/j.molcel.2015.03.030 PMC4475440

[32] Jeronimo, C. & Robert, F. The histone chaperone FACT: a guardian of chromatin structure integrity. *Transcription* **13**, 16–38 (2022). 35485711 10.1080/21541264.2022.2069995 PMC9467567

[33] Svensson, J. P. et al. A nucleosome turnover map reveals that the stability of histone H4 Lys20 methylation depends on histone recycling in transcribed chromatin. *Genome Res.* **25**, 872–883 (2015). 25778913 10.1101/gr.188870.114 PMC4448683

[34] Žumer, K. et al. FACT maintains chromatin architecture and thereby stimulates RNA polymerase II pausing during transcription in vivo. *Mol. Cell* **84**, 2053–2069 (2024). 38810649 10.1016/j.molcel.2024.05.003

[35] Kujirai, T. et al. Structural basis of the nucleosome transition during RNA polymerase II passage. *Science* **362**, 595–598 (2018). 30287617 10.1126/science.aau9904

[36] Farnung, L., Vos, S. M. & Cramer, P. Structure of transcribing RNA polymerase II-nucleosome complex. *Nat. Commun.* **9**, 5432 (2018). 30575770 10.1038/s41467-018-07870-y PMC6303367

[37] Kizer, K. O. et al. A Novel Domain in Set2 Mediates RNA Polymerase II Interaction and Couples Histone H3 K36 Methylation with Transcript Elongation. *Mol. Cell. Biol.* **25**, 3305–3316 (2005). 15798214 10.1128/MCB.25.8.3305-3316.2005 PMC1069628

[38] Li, B., Howe, L., Anderson, S., Yates, J. R. & Workman, J. L. The set2 histone methyltransferase functions through the phosphorylated carboxyl-terminal domain of RNA polymerase II. *J. Biol. Chem.* **278**, 8897–8903 (2003). 12511561 10.1074/jbc.M212134200

[39] Li, J., Moazed, D. & Gygi, S. P. Association of the histone methyltransferase set2 with RNA polymerase II plays a role in transcription elongation. *J. Biol. Chem.* **277**, 49383–49388 (2002). 12381723 10.1074/jbc.M209294200

[40] Xiao, T. et al. Phosphorylation of RNA polymerase II CTD regulates H3 methylation in yeast. *Genes Dev.* **17**, 654–663 (2003). 12629047 10.1101/gad.1055503 PMC196010

[41] Morris, S. A. et al. Histone H3 K36 methylation Is associated with transcription elongation in Schizosaccharomyces pombe. *Eukaryot. Cell* **4**, 1446–1454 (2005). 16087749 10.1128/EC.4.8.1446-1454.2005 PMC1214526

[42] Krogan, N. J. et al. Methylation of histone H3 by Set2 in Saccharomyces cerevisiae Is Linked to Transcriptional Elongation by RNA Polymerase II. *Mol. Cell. Biol.* **23**, 4207–4218 (2003). 12773564 10.1128/MCB.23.12.4207-4218.2003 PMC427527

[43] Keogh, M.-C. et al. Cotranscriptional Set2 methylation of histone H3 lysine 36 recruits a repressive Rpd3 complex. *Cell* **123**, 593–605 (2005). 16286008 10.1016/j.cell.2005.10.025

[44] Li, B. et al. Combined action of PHD and chromo domains directs the Rpd3S HDAC to transcribed chromatin. *Science* **316**, 1050–1054 (2007). 17510366 10.1126/science.1139004

[45] Musselman, C. A. et al. Molecular basis for H3K36me3 recognition by the Tudor domain of PHF1. *Nat. Struct. Mol. Biol.* **19**, 1266–1272 (2012). 23142980 10.1038/nsmb.2435 PMC3603146

[46] Rona, G. B., Eleutherio, E. C. A. & Pinheiro, A. S. PWWP domains and their modes of sensing DNA and histone methylated lysines. *Biophys. Rev.* **8**, 63–74 (2016). 28510146 10.1007/s12551-015-0190-6 PMC5425739

[47] Jha, D. K. & Strahl, B. D. An RNA polymerase II-coupled function for histone H3K36 methylation in checkpoint activation and DSB repair. *Nat. Commun.* **5**, 3965 (2014). 24910128 10.1038/ncomms4965 PMC4052371

[48] Molenaar, T. M. & van Leeuwen, F. SETD2: from chromatin modifier to multipronged regulator of the genome and beyond. *Cell. Mol. Life Sci.* **79**, 346 (2022). 35661267 10.1007/s00018-022-04352-9 PMC9167812

[49] Zhu, X. et al. Identification of functional cooperative mutations of SETD2 in human acute leukemia. *Nat. Genet.* **46**, 287–293 (2014). 24509477 10.1038/ng.2894 PMC4440318

[50] Dalgliesh, G. L. et al. Systematic sequencing of renal carcinoma reveals inactivation of histone modifying genes. *Nature* **463**, 360–363 (2010). 20054297 10.1038/nature08672 PMC2820242

[51] Sun, X.-J. et al. Identification and characterization of a novel human histone H3 lysine 36-specific methyltransferase. *J. Biol. Chem.* **280**, 35261–35271 (2005). 16118227 10.1074/jbc.M504012200

[52] Vojnic, E., Simon, B., Strahl, B. D., Sattler, M. & Cramer, P. Structure and carboxyl-terminal domain (CTD) binding of the Set2 SRI domain that couples histone H3 Lys36 methylation to transcription. *J. Biol. Chem.* **281**, 13–15 (2006). 16286474 10.1074/jbc.C500423200

[53] Li, M. et al. Solution structure of the Set2–Rpb1 interacting domain of human Set2 and its interaction with the hyperphosphorylated C-terminal domain of Rpb1. *Proc. Natl. Acad. Sci. USA* **102**, 17636–17641 (2005). 16314571 10.1073/pnas.0506350102 PMC1308900

[54] Wang, Y., Niu, Y. & Li, B. Balancing acts of SRI and an auto-inhibitory domain specify Set2 function at transcribed chromatin. *Nucleic Acids Res.* **43**, 4881–4892 (2015). 25925577 10.1093/nar/gkv393 PMC4446442

[55] Gopalakrishnan, R., Marr, S. K., Kingston, R. E. & Winston, F. A conserved genetic interaction between Spt6 and Set2 regulates H3K36 methylation. *Nucleic Acids Res.* **47**, 3888–3903 (2019). 30793188 10.1093/nar/gkz119 PMC6486648

[56] Youdell, M. L. et al. Roles for Ctk1 and Spt6 in regulating the different methylation states of histone H3 lysine 36. *Mol. Cell. Biol.* **28**, 4915–4926 (2008). 18541663 10.1128/MCB.00001-08 PMC2519698

[57] Zheng, W. et al. Sinefungin derivatives as inhibitors and structure probes of protein lysine methyltransferase SETD2. *J. Am. Chem. Soc.* **134**, 18004–18014 (2012). 23043551 10.1021/ja307060p PMC3504124

[58] Liu, Y. et al. Cryo-EM structure of SETD2/Set2 methyltransferase bound to a nucleosome containing oncohistone mutations. *Cell Discov.* **7**, 1–12 (2021). 33972509 10.1038/s41421-021-00261-6 PMC8110526

[59] Mirdita, M. et al. ColabFold: making protein folding accessible to all. *Nat. Methods* **19**, 679–682 (2022). 35637307 10.1038/s41592-022-01488-1 PMC9184281

[60] Jumper, J. et al. Highly accurate protein structure prediction with AlphaFold. *Nature* **596**, 583–589 (2021). 34265844 10.1038/s41586-021-03819-2 PMC8371605

[61] Kemble, D. J., McCullough, L. L., Whitby, F. G., Formosa, T. & Hill, C. P. FACT Disrupts nucleosome structure by binding H2A-H2B with conserved peptide motifs. *Mol. Cell* **60**, 294–306 (2015). 26455391 10.1016/j.molcel.2015.09.008 PMC4620744

[62] Liu, Y. et al. FACT caught in the act of manipulating the nucleosome. *Nature* **577**, 426–431 (2020). 31775157 10.1038/s41586-019-1820-0 PMC7441595

[63] Mayanagi, K. et al. Structural visualization of key steps in nucleosome reorganization by human FACT. *Sci. Rep.* **9**, 10183 (2019). 31308435 10.1038/s41598-019-46617-7 PMC6629675

[64] Bortvin, A. & Winston, F. Evidence that Spt6p controls chromatin structure by a direct interaction with histones. *Science* **272**, 1473–1476 (1996). 8633238 10.1126/science.272.5267.1473

[65] Miller, C. L. W., Warner, J. L. & Winston, F. Insights into Spt6: a histone chaperone that functions in transcription, DNA replication, and genome stability. *Trends Genet.* **39**, 858–872 (2023). 37481442 10.1016/j.tig.2023.06.008 PMC10592469

[66] Bilokapic, S. & Halic, M. Nucleosome and ubiquitin position Set2 to methylate H3K36. *Nat. Commun.* **10**, 1–9 (2019). 31439846 10.1038/s41467-019-11726-4 PMC6706414

[67] Li, Y. & Huang, H. Structural basis for H2A–H2B recognitions by human Spt16. *Biochem. Biophys. Res. Commun.* **651**, 85–91 (2023). 36801613 10.1016/j.bbrc.2023.02.016

[68] Nakane, T., Kimanius, D., Lindahl, E. & Scheres, S. H. Characterisation of molecular motions in cryo-EM single-particle data by multi-body refinement in RELION. *ELife* **7**, e36861 (2018). 29856314 10.7554/eLife.36861 PMC6005684

[69] Hornbeck, P. V. et al. PhosphoSitePlus, 2014: mutations, PTMs and recalibrations. *Nucleic Acids Res.* **43**, D512–D520 (2015). 25514926 10.1093/nar/gku1267 PMC4383998

[70] Bhattacharya, S. et al. The disordered regions of the methyltransferase SETD2 govern its function by regulating its proteolysis and phase separation. *J. Biol. Chem.* **297**, 101075 (2021). 34391778 10.1016/j.jbc.2021.101075 PMC8405934

[71] Fuchs, S. M., Kizer, K. O., Braberg, H., Krogan, N. J. & Strahl, B. D. RNA Polymerase II carboxyl-terminal domain phosphorylation regulates protein stability of the set2 methyltransferase and histone H3 Di- and trimethylation at lysine 36. *J. Biol. Chem.* **287**, 3249–3256 (2012). 22157004 10.1074/jbc.M111.273953 PMC3270979

[72] Khan, A. et al. A SETD2–CDK1–lamin axis maintains nuclear morphology and genome stability. *Nat. Cell Biol.* **27**, 1327–1341 (2025). 40789955 10.1038/s41556-025-01723-9 PMC12912276

[73] Markert, J. W., Soffers, J. H. & Farnung, L. Structural basis of H3K36 trimethylation by SETD2 during chromatin transcription. *Science* **387**, 528–533 (2025). 39666822 10.1126/science.adn6319 PMC12366524

[74] Kujirai, T. et al. Structural basis of transcription-coupled H3K36 trimethylation by Set2 and RNAPII elongation complex in the nucleosome. Preprint at 10.1101/2024.12.13.628464 (2024).

[75] Zhang, S. et al. Structure of a transcribing RNA polymerase II–U1 snRNP complex. *Science* **371**, 305–309 (2021). 33446560 10.1126/science.abf1870

[76] Li, Y., Wang, Q., Xu, Y. & Li, Z. Structures of co-transcriptional RNA capping enzymes on paused transcription complex. *Nat. Commun.* **15**, 4622 (2024). 38816438 10.1038/s41467-024-48963-1 PMC11139899

[77] Garg, G. et al. Structural insights into human co-transcriptional capping. *Mol. Cell* **83**, 2464–2477 (2023). 37369200 10.1016/j.molcel.2023.06.002

[78] Talbert, P. B. & Henikoff, S. The Yin and Yang of histone marks in transcription. *Annu. Rev. Genomics Hum. Genet.* **22**, 147–170 (2021). 33781079 10.1146/annurev-genom-120220-085159

[79] Xiao, T. et al. Histone H2B Ubiquitylation Is Associated with Elongating RNA Polymerase II. *Mol. Cell. Biol.* **25**, 637–651 (2005). 15632065 10.1128/MCB.25.2.637-651.2005 PMC543430

[80] Bae, H. J. et al. The Set1 N-terminal domain and Swd2 interact with RNA polymerase II CTD to recruit COMPASS. *Nat. Commun.* **11**, 2181 (2020). 32358498 10.1038/s41467-020-16082-2 PMC7195483

[81] Kim, S.-K. et al. Human histone H3K79 methyltransferase DOT1L methyltransferase binds actively transcribing RNA polymerase II to regulate gene expression. *J. Biol. Chem.* **287**, 39698–39709 (2012). 23012353 10.1074/jbc.M112.384057 PMC3501035

[82] Levendosky, R. F., Sabantsev, A., Deindl, S. & Bowman, G. D. The Chd1 chromatin remodeler shifts hexasomes unidirectionally. *ELife* **5**, e21356 (2016). 28032848 10.7554/eLife.21356 PMC5226652

[83] Dyer, P. N. et al. in* Methods in Enzymology.* (Elsevier, 2003).

[84] Dombrowski, M., Engeholm, M., Dienemann, C., Dodonova, S. & Cramer, P. Histone H1 binding to nucleosome arrays depends on linker DNA length and trajectory. *Nat. Struct. Mol. Biol.* **29**, 493–501 (2022). 35581345 10.1038/s41594-022-00768-w PMC9113941

[85] Mastronarde, D. N. Automated electron microscope tomography using robust prediction of specimen movements. *J. Struct. Biol.* **152**, 36–51 (2005). 16182563 10.1016/j.jsb.2005.07.007

[86] Tegunov, D. & Cramer, P. Real-time cryo-electron microscopy data preprocessing with Warp. *Nat. Methods* **16**, 1146–1152 (2019). 31591575 10.1038/s41592-019-0580-y PMC6858868

[87] Scheres, S. H. W. RELION: Implementation of a Bayesian approach to cryo-EM structure determination. *J. Struct. Biol.* **180**, 519–530 (2012). 23000701 10.1016/j.jsb.2012.09.006 PMC3690530

[88] Punjani, A., Rubinstein, J. L., Fleet, D. J. & Brubaker, M. A. cryoSPARC: algorithms for rapid unsupervised cryo-EM structure determination. *Nat. Methods* **14**, 290–296 (2017). 28165473 10.1038/nmeth.4169

[89] Zivanov, J. et al. New tools for automated high-resolution cryo-EM structure determination in RELION-3. *ELife* **7**, e42166 (2018). 30412051 10.7554/eLife.42166 PMC6250425

[90] Kimanius, D. et al. Data-driven regularization lowers the size barrier of cryo-EM structure determination. *Nat. Methods* **21**, 1216–1221 (2024). 38862790 10.1038/s41592-024-02304-8 PMC11239489

[91] Croll, T. I. *ISOLDE*: a physically realistic environment for model building into low-resolution electron-density maps. *Acta Crystallogr. Sect. Struct. Biol.* **74**, 519–530 (2018). 10.1107/S2059798318002425 PMC6096486 29872003

[92] Tsunaka, Y. Alteration of the nucleosomal DNA path in the crystal structure of a human nucleosome core particle. *Nucleic Acids Res.* **33**, 3424–3434 (2005). 15951514 10.1093/nar/gki663 PMC1150222

[93] Goddard, T. D. et al. UCSF ChimeraX: Meeting modern challenges in visualization and analysis. *Protein Sci.* **27**, 14–25 (2018). 28710774 10.1002/pro.3235 PMC5734306

[94] Pettersen, E. F. et al. UCSF ChimeraX: Structure visualization for researchers, educators, and developers. *Protein Sci.* **30**, 70–82 (2021). 32881101 10.1002/pro.3943 PMC7737788

[95] Emsley, P. & Cowtan, K. Coot: model-building tools for molecular graphics. *Acta Crystallogr. D Biol. Crystallogr.* **60**, 2126–2132 (2004). 15572765 10.1107/S0907444904019158

[96] Adams, P. D. et al. *PHENIX*: a comprehensive Python-based system for macromolecular structure solution. *Acta Crystallogr. D Biol. Crystallogr.* **66**, 213–221 (2010). 20124702 10.1107/S0907444909052925 PMC2815670

[97] Afonine, P. V. et al. Real-space refinement in *PHENIX* for cryo-EM and crystallography. *Acta Crystallogr. Sect. Struct. Biol.* **74**, 531–544 (2018). 10.1107/S2059798318006551 PMC6096492 29872004

[98] Yang, B. et al. Identification of cross-linked peptides from complex samples. *Nat. Methods* **9**, 904–906 (2012). 22772728 10.1038/nmeth.2099

[99] Chen, Z.-L. et al. A high-speed search engine pLink 2 with systematic evaluation for proteome-scale identification of cross-linked peptides. *Nat. Commun.* **10**, 3404 (2019). 31363125 10.1038/s41467-019-11337-z PMC6667459

[100] Combe, C. W., Fischer, L. & Rappsilber, J. xiNET: Cross-link Network Maps With Residue Resolution*. *Mol. Cell. Proteom.* **14**, 1137–1147 (2015). 10.1074/mcp.O114.042259 PMC4390258 25648531

