# Supplementary material for: Molecular mechanism of co-transcriptional H3K36 methylation by SETD2
Source: Nat Commun. 2025 Oct 29;16:9565. doi: 10.1038/s41467-025-65439-y (PMC12572195; doi:10.1038/s41467-025-65439-y)
Supplement: Supplementary file 2 — Description of additional supplementary files [file 41467_2025_65439_MOESM2_ESM.pdf]

# Description of Additional Supplementary Files

File Name: Supplementary Data 1

Description: Supplementary Data 1: Protein-protein crosslinks identified in the mammalian activated elongation complex in the presence of SETD2 and FACT. Numbers in the "Residue 1" and Residue 2" columns indicate the position of the crosslinked lysine or N-terminal residue in the protein 1 and protein 2, respectively. Crosslinked peptide spectrum matches (CSMs) and the score (as a negative logarithm of the pLink Score) are shown. Higher values in the "CSMs" and "Score" correspond to more confident identifications. Only crosslinks that passed a filter of false discovery rate of 5% are listed.
