# Supplementary material for: Molecular mechanism of co-transcriptional H3K36 methylation by SETD2
Source: Nat Commun. 2025 Oct 29;16:9565. doi: 10.1038/s41467-025-65439-y (PMC12572195; doi:10.1038/s41467-025-65439-y)
Supplement: Supplementary file 1 — Supplementary Information [file 41467_2025_65439_MOESM1_ESM.pdf]

Molecular mechanism of co-transcriptional H3K36 methylation by SETD2

James L. Walshe[1*#], Moritz Ochmann[1*], Ute Neef[1], Olexandr Dybkov[2], Christian Dienemann[1], Christiane Oberthür[1], Aiturgan Zheenbekova[1], Henning Urlaub[2,3], Patrick Cramer[1#]

[1] Max Planck Institute for Multidisciplinary Sciences, Department of Molecular Biology, Am Fassberg 11, 37077 Göttingen, Germany.
[2] Bioanalytical Mass Spectrometry, Max Planck Institute for Multidisciplinary Sciences, 37077 Göttingen, Germany.
[3] Institute of Clinical Chemistry, Bioanalytics Group, University Medical Center Göttingen, 37075 Göttingen, Germany.

*These authors contributed equally
#Correspondence: james.walshe@mpinat.mpg.de, patrick.cramer@mpinat.mpg.de

Supplementary Figures 1 - 19

Supplementary Tables 1 - 3

Supplementary Figure 1

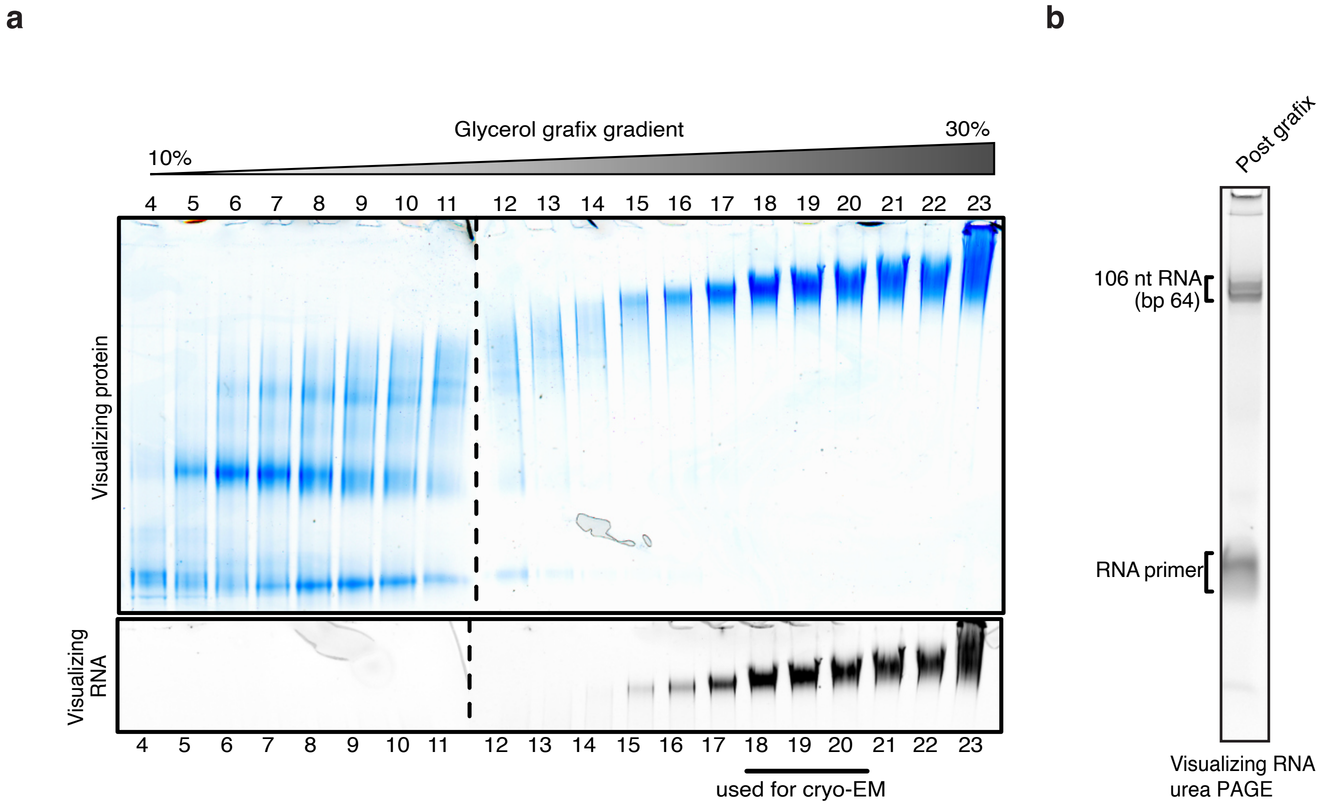

Supplementary Figure 1: Cryo-EM sample preparation of State 1 complex.

a, Native PAGE gel of the 10-30% grafix glycerol gradient fractions of the methylation competent elongation complex transcribed to nucleosomal bp 64. In the top gel image, proteins are visualized using coomassie staining. The bottom gel image visualizes the 5'Cy5 labelled RNA. Fractions used for cryo-EM analysis are indicated.
b, Denaturing PAGE of complex formation for cryo-EM analysis to verify RNA extension to nucleosomal bp 64. The 5'Cy5 label of the RNA was visualized. Source data are provided as a Source Data file.

Supplementary Figure 2

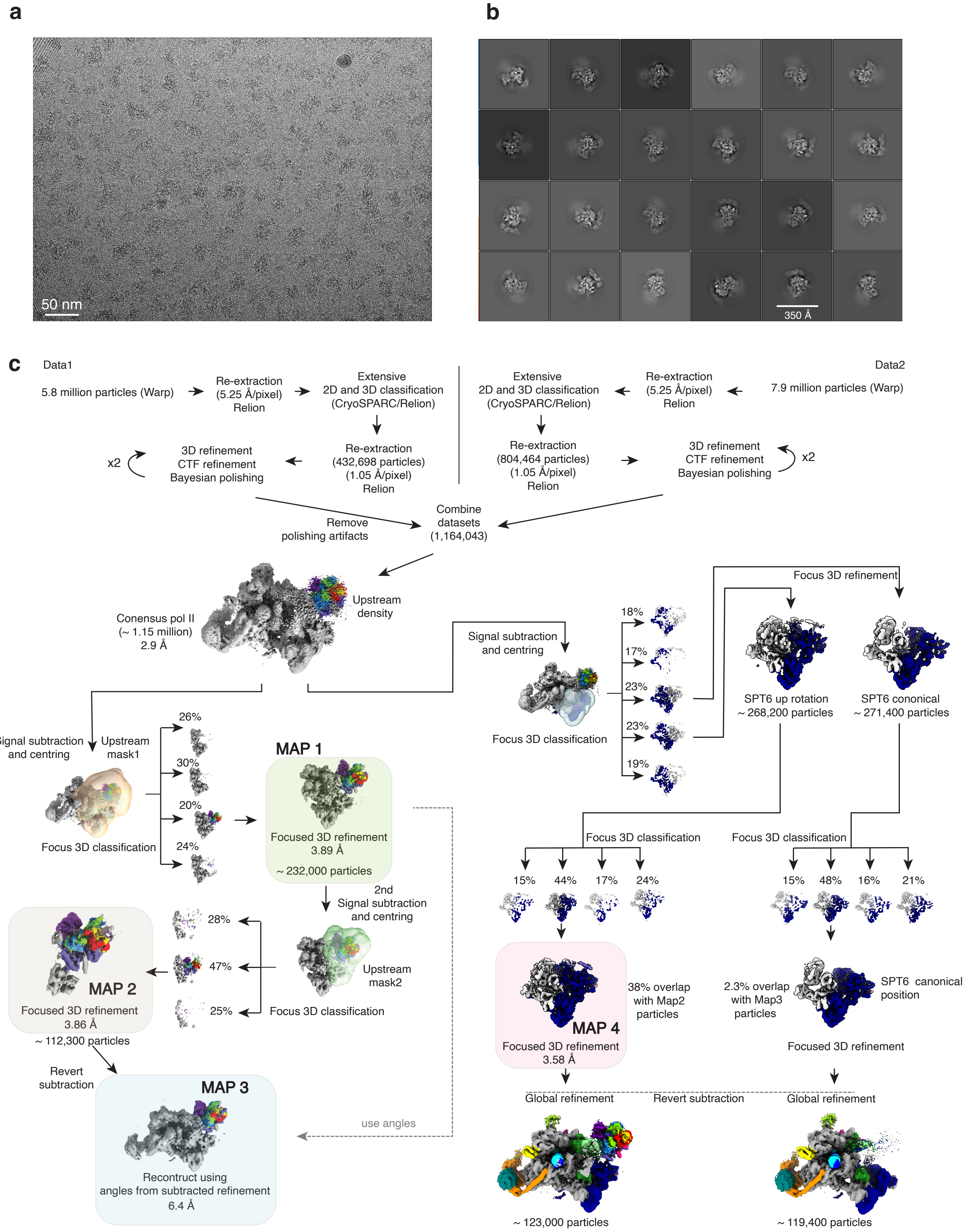

Supplementary Figure 2: Data acquisition and processing of the State 1 complex.

a, Representative micrograph of the State 1 data collection with scale bar.
b, Representative 2D classes of mammalian activated elongation complex obtained during classification.
c, Processing and classification strategy employed to classify the methylation competent elongation complex from the recorded micrographs. Major steps are described within the figure. Coulomb maps used to build models are highlighted.

Supplementary Figure 3

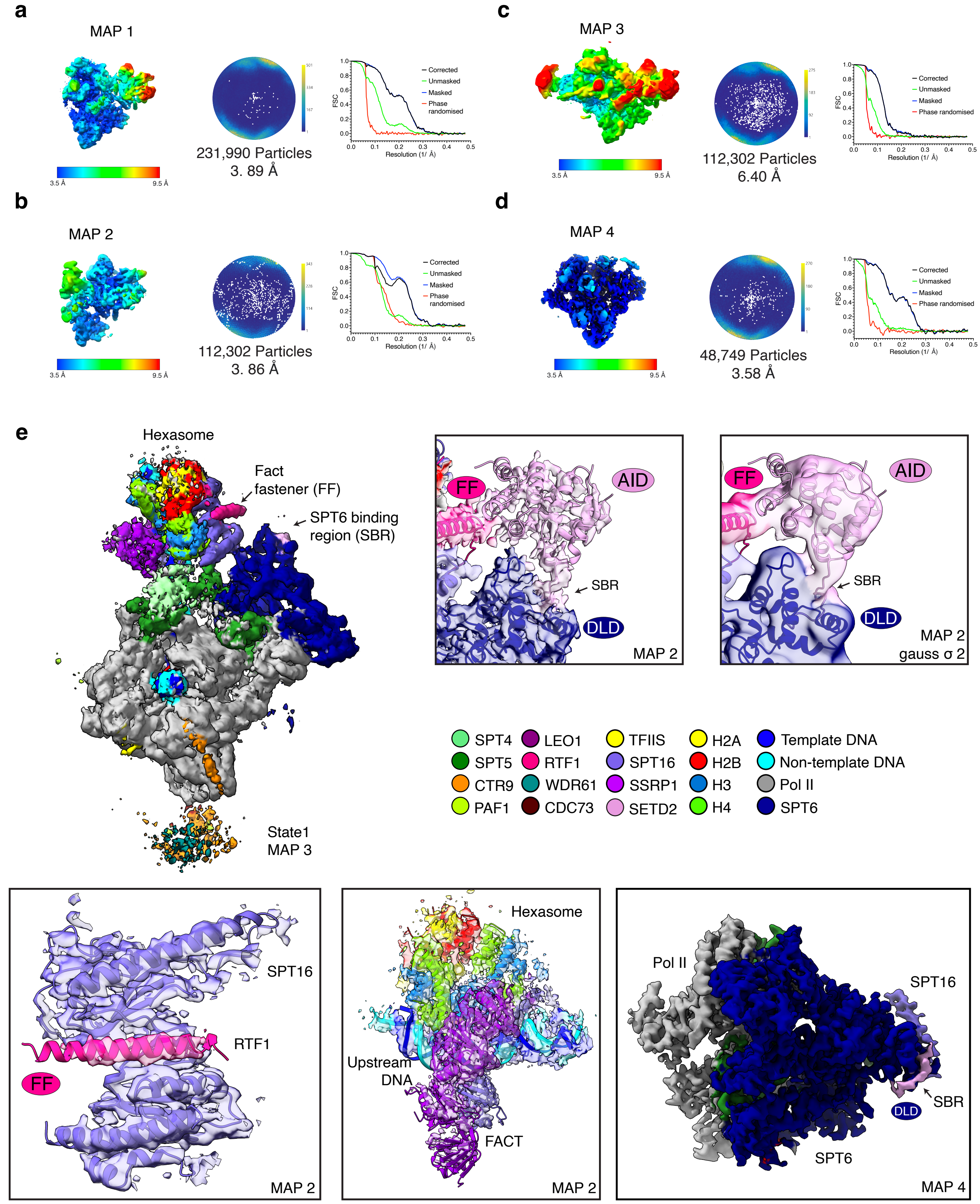

Supplementary Figure 3: Cryo-EM data metrics of State 1 complex and map quality.

a-d, Reconstructions of MAP1-4 obtained from the State 1 dataset coloured by their local resolution as estimated using RELION. TRELION. Total particle count, global resolution estimate using the Fourier shell correlation (FSC) = 0.143 criterion, and angular distribution plot are shown.
e, Various reconstructions highlighting data quality. Map number indicated in figure.

Supplementary Figure 4

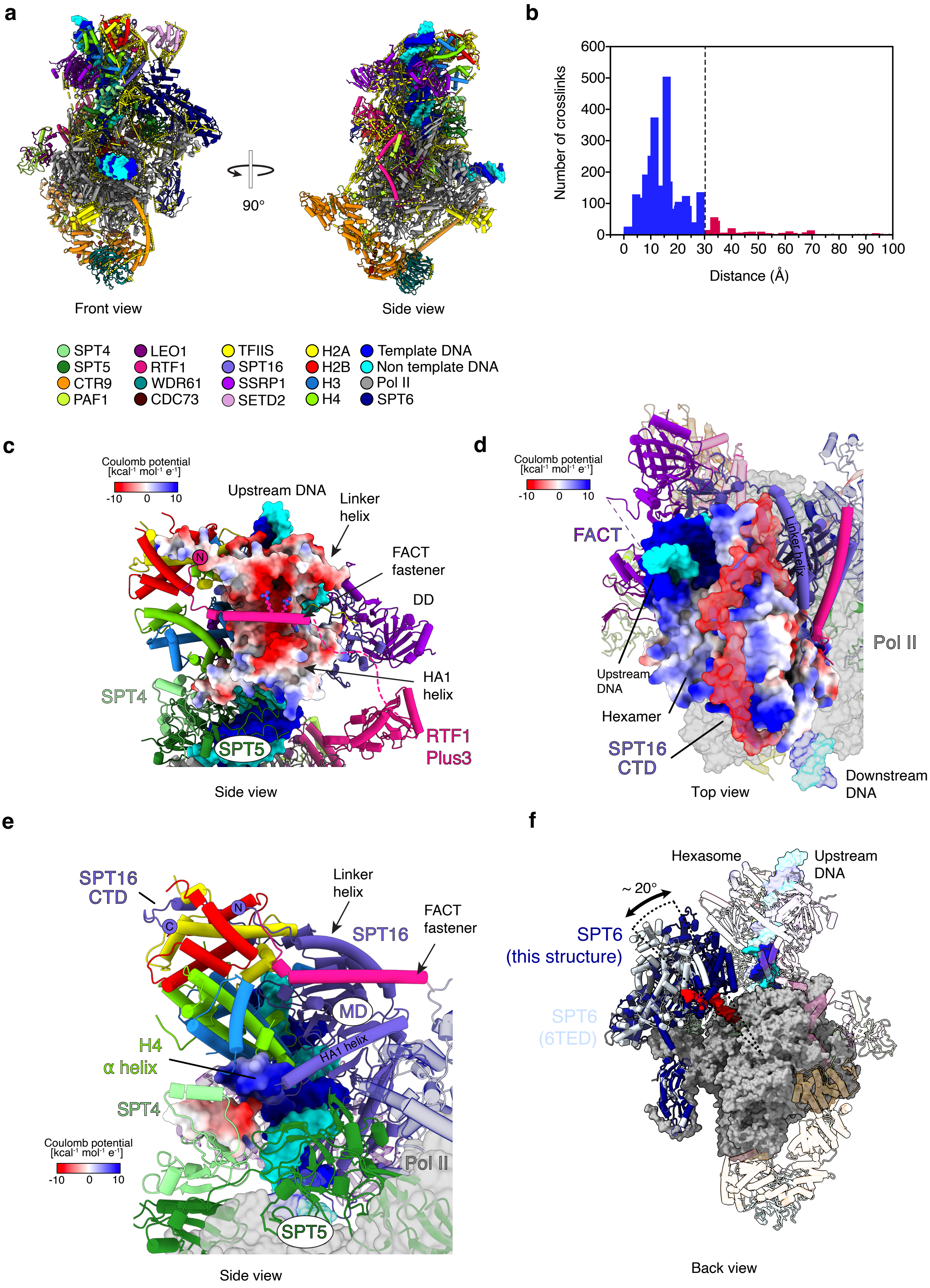

Supplementary Figure 4: Chemical cross-linking mass spectrometry and State 1 interactions.

a, Front and side view of State 1 with mapped BS3 crosslinks (cutoff >30 Å).
b, Histogram of crosslink counts and the distances between Nζ pairs that were mapped onto the model. In cases where the side chain was not modelled, Cα pairs were used. 9 crosslinks with a distance longer than 100 Å were excluded from the histogram.
c, SPT16 CTD binds exposed histones. SPT16 is shown by its coulombic surface potential. Arg 289, Arg 292 and Arg 295 in the RTF1 FACT "fastener" represented as spheres.
d, Top view of upstream hexasome in State 1. Histones and SPT16 CTD are shown by their coulombic surface potential.
e, Interaction of DSIF with histones and SPT16. SPT4 interacts with α1 helix of H4 (both partially presented by their coulombic surface potential). SPT5 contacts SPT16's HA1 helix and MD.
f, SPT6 rearranges to contact the upstream hexasome bound by FACT. Structural comparison of the activated elongation complex (PDB: 6TED – coloured light steel blue)[1] reveals significant rotation (~20°) and rearrangement of SPT6 to contact the upstream hexasome. State 1 SPT6 shown in dark blue. Other elongation factors and the upstream hexasome with FACT are represented by transparent cartoons.

Supplementary Figure 5

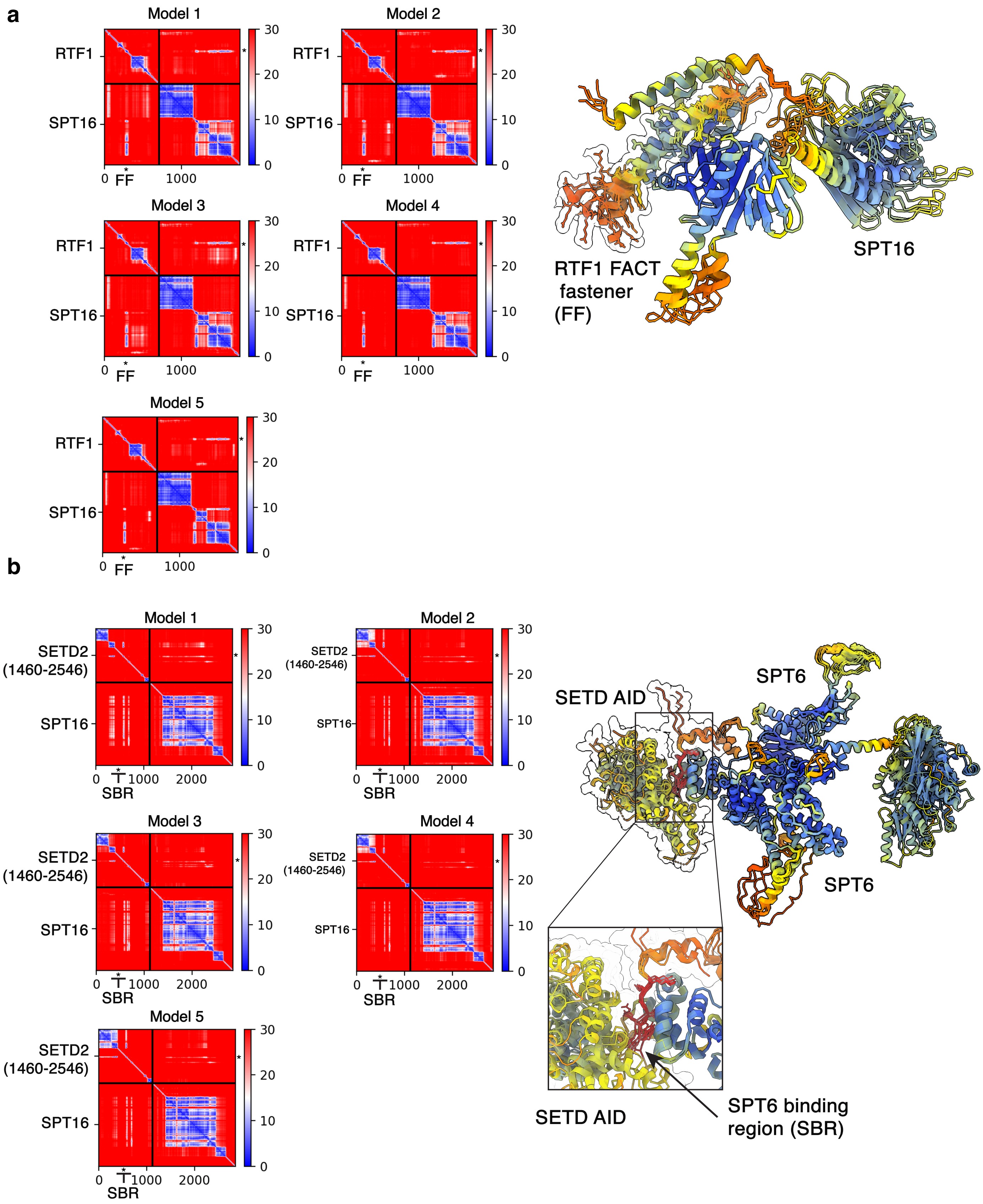

Supplementary Figure 5: ColabFold[2] prediction of protein interactions.

a, Left - Predicted aligned error (PAE) of full-length RTF1 and SPT16. Predicted FACT “fastener” interaction marked with an asterisk. Right - Overlay of the 5 predicted models of SPT16 (residues 466-929 shown) and RTF1 (residues 266-304 shown). Cartoon colored by pLDDT confidence.
b, Left - Predicted aligned error (PAE) of full-length SETD2 (residues 1460-2546) and SPT6. Predicted SPT6 binding region interaction marked with an asterisk. Right - Overlay of the 5 predicted models of SPT6 (residues 1731-1852,2002-2059 shown) and SPT6 (residues 266-1521 shows). Cartoon colored by pLDDT confidence.

Supplementary Figure 6

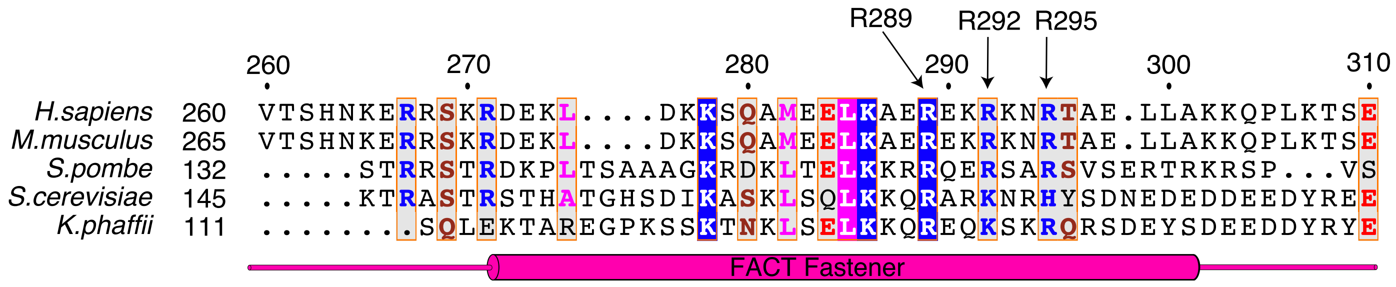

Supplementary Figure 6: RTF1 FACT “fastener” sequence alignment.

Sequence alignment of the FACT “fastener” from RTF1 generated using ClustalOmega[3] and visualized in ESPript[4]. Sequences from *Homo sapiens*, *Mus musculus*, *Schizosaccharomyces pombe*, *Saccharomyces cerevisiae* and *Komagataella phaffii* were aligned and compared. The FACT “fastener” of RTF1 and the positive charge residues are highly conserved. Arg289, Arg292 and Arg295 of the FACT “fastener” are marked by arrows.

Supplementary Figure 7

a

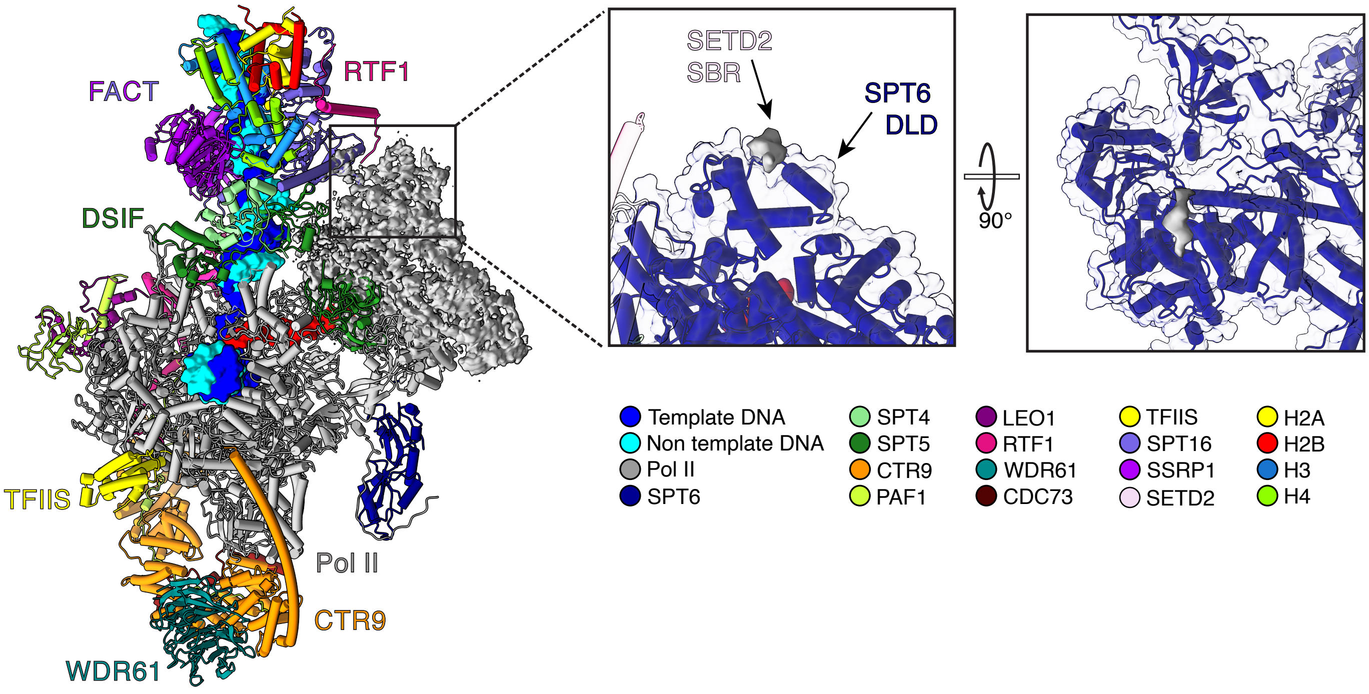

Top view

b

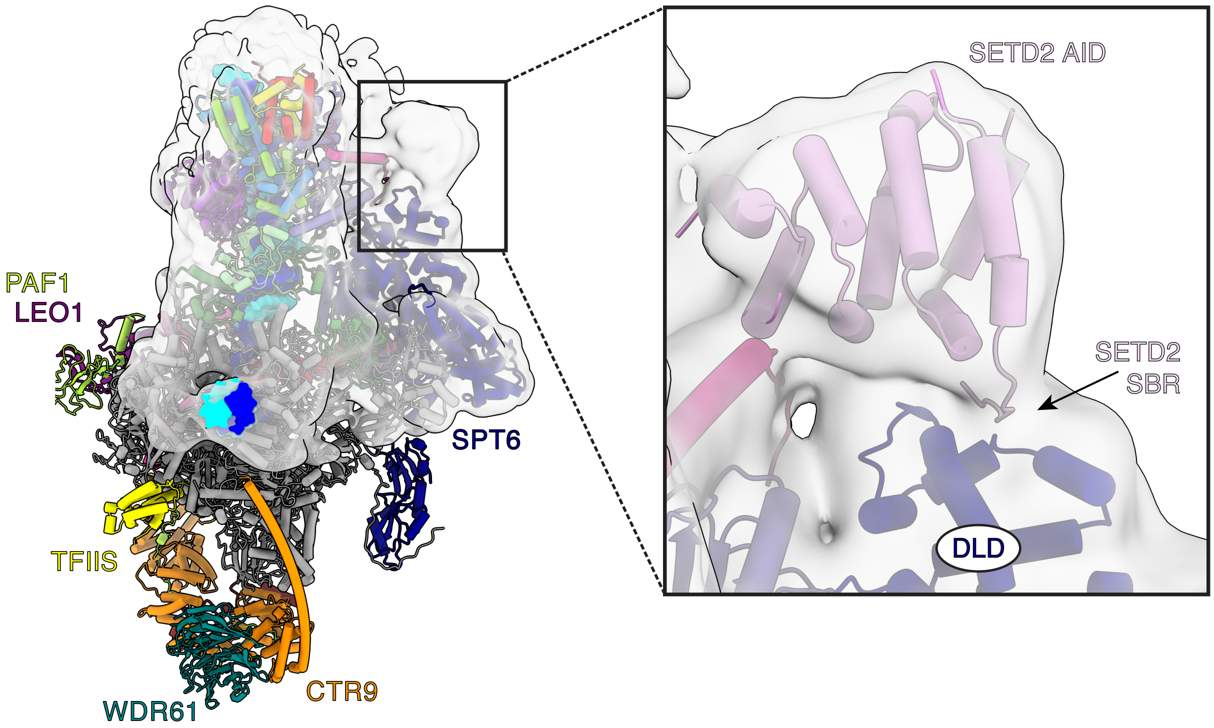

Top view

Supplementary Figure 7: SETD2 binds SPT6 dealth-like domain (DLD).

a, Top view of State 1 with cryo-EM density of MAP 4 (Supplementary Figure 3d) displayed in grey. Cryo-EM density for the modelled region the SETD2 SPT6 binding region (SBR) shown in zoomed panels.
b, Top view of State 1 with the gaussian filtered (σ = 2) cryo-EM density of MAP 1 (Supplementary Figure 3b) shown in grey. Alphafold2 model for SETD2 AID shown in panel and rigid body fitted into density. Interaction between SPT6 DLD and SETD2 modelled based of ColabFold predictions (Supplementary Figure 5).

Supplementary Figure 8

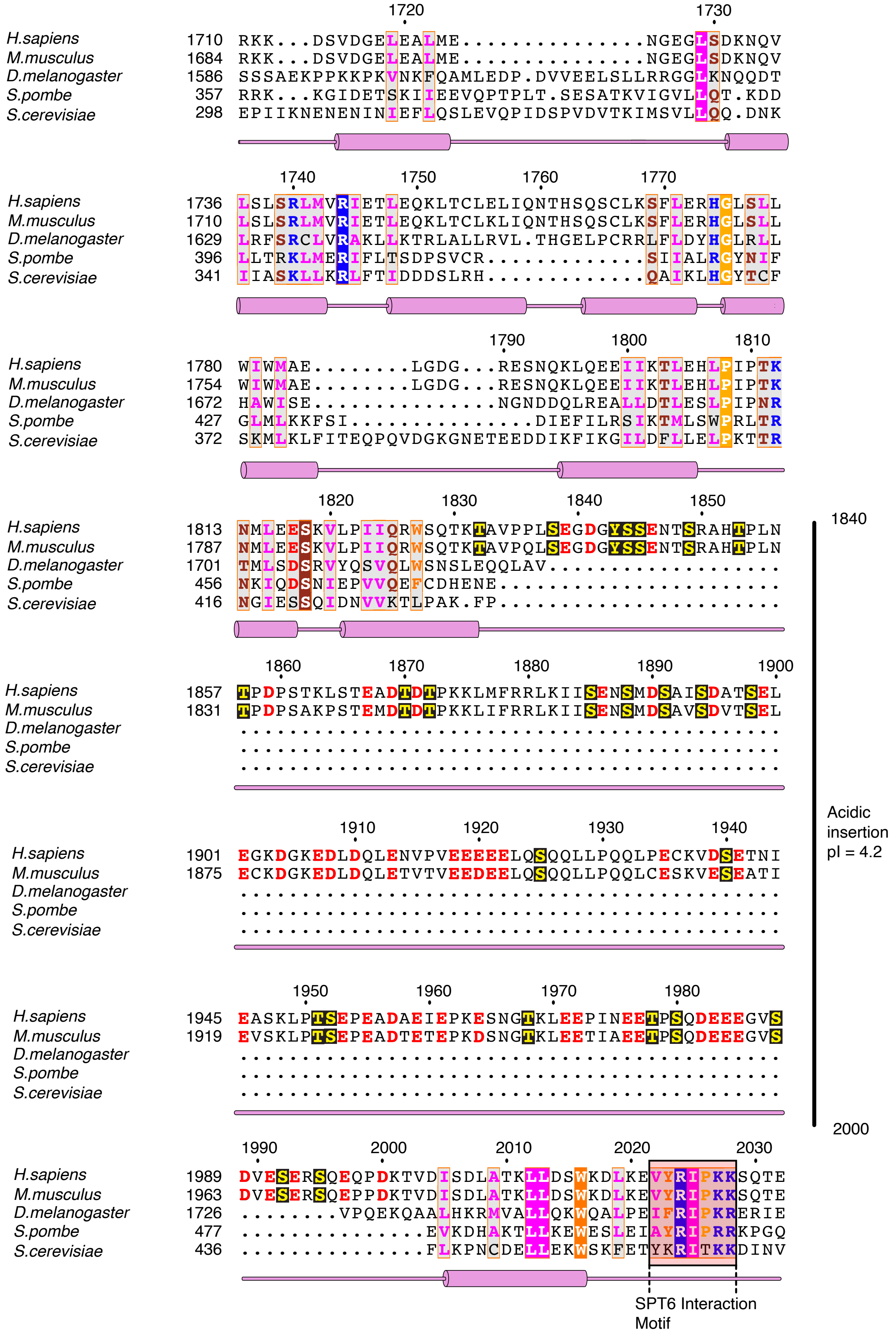

Supplementary Figure 8: SETD2 SPT6 binding region and acidic insertion sequence alignment.

Sequence alignment of the SETD2 SPT6 binding region generated using ClustalOmega[3] and visualized in ESPript[4]. Sequences from *Homo sapiens*, *Mus musculus*, *Schizosaccharomyces pombe*, *Saccharomyces cerevisiae* and *Drosophila melanogaster* were aligned and compared. Resides 2022 – 2028 are highly conserved. Residues within the acidic insertion predicted to undergo phosphorylation are highlighted in yellow on black background[5].

Supplementary Figure 9

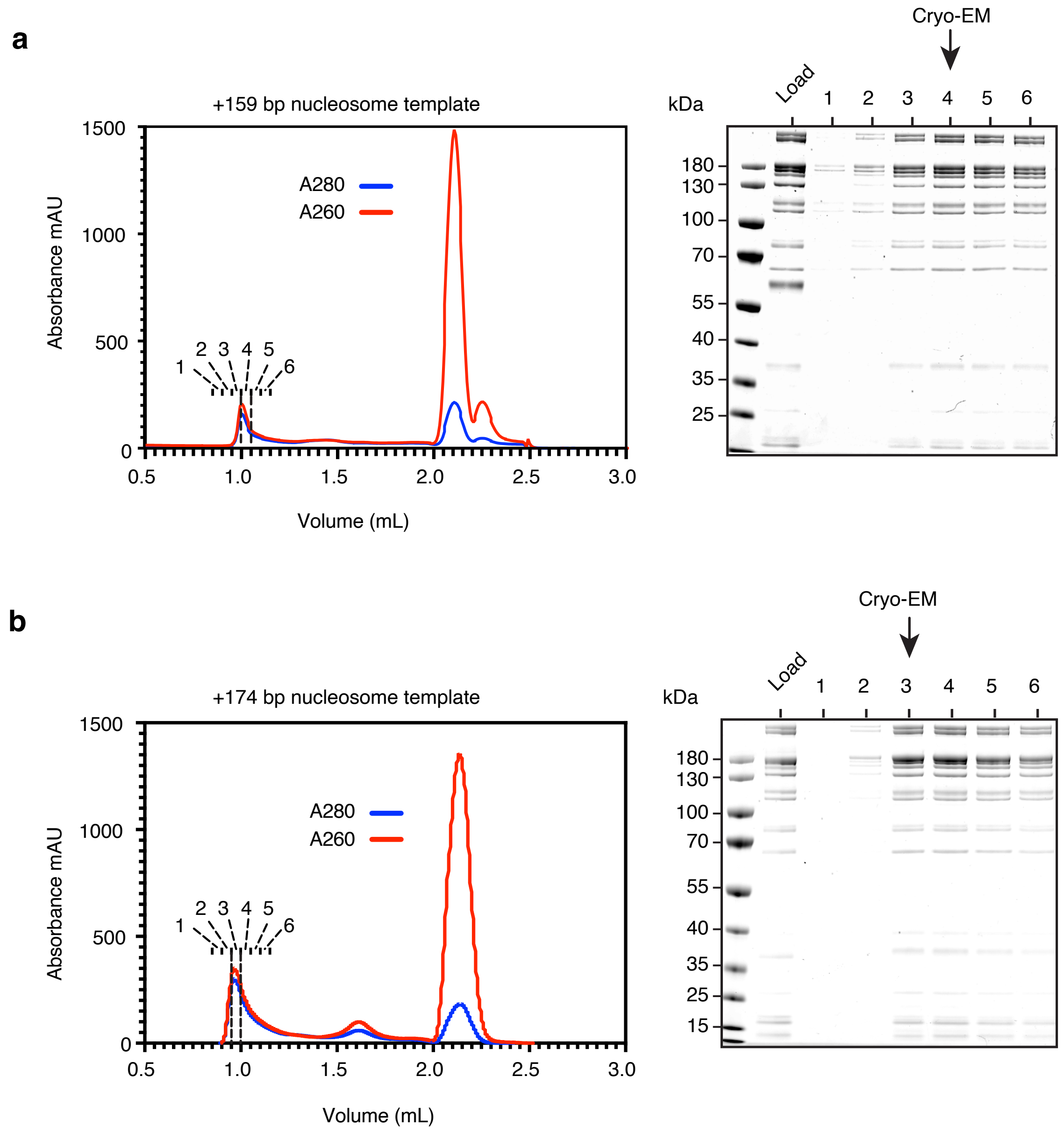

Supplementary Figure 9: Cryo-EM sample purification of State 2 and State 3 complexes.

a, Left - Chromatogram of Superose 6 Increase (3.2/300) purification of methylation competent elongation complex bound to a ligated nucleosome template that would represent transcription to bp 159 into the nucleosome template (State 2). Right - SDS-PAGE analysis of peak fractions. Fraction used for cryo-EM analysis is indicated. Source data are provided as a Source Data file.

b, Left - Chromatogram of Superose 6 Increase (3.2/300) purification of methylation competent elongation complex bound to a ligated nucleosome template that would represent transcription to bp 174 into the nucleosome template (State 3). Right - SDS-PAGE analysis of peak fractions. Fraction used for cryo-EM analysis is indicated. Source data are provided as a Source Data file.

Supplementary Figure 10

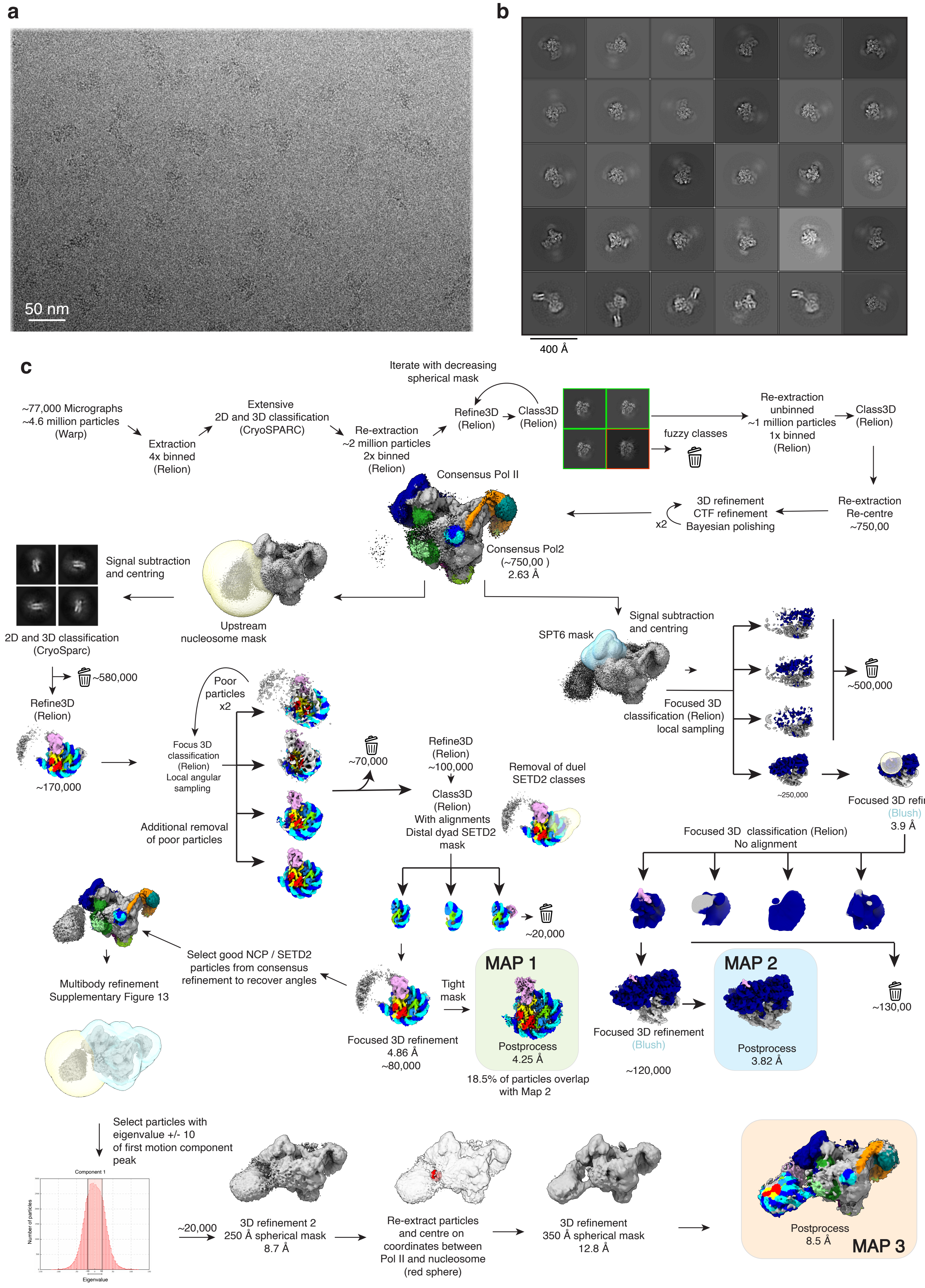

Supplementary Figure 10: Data acquisition and processing of State 2 complex.

a, Representative micrograph of the State 2 data collection with scale bar.
b, Representative 2D classes of mammalian activated elongation complex obtained during classification.
c, Processing and classification strategy employed to classify the methylation competent elongation complex from obtained micrographs. Major steps are described within the figure. Coulomb maps used to build models are highlighted.

Supplementary Figure 11

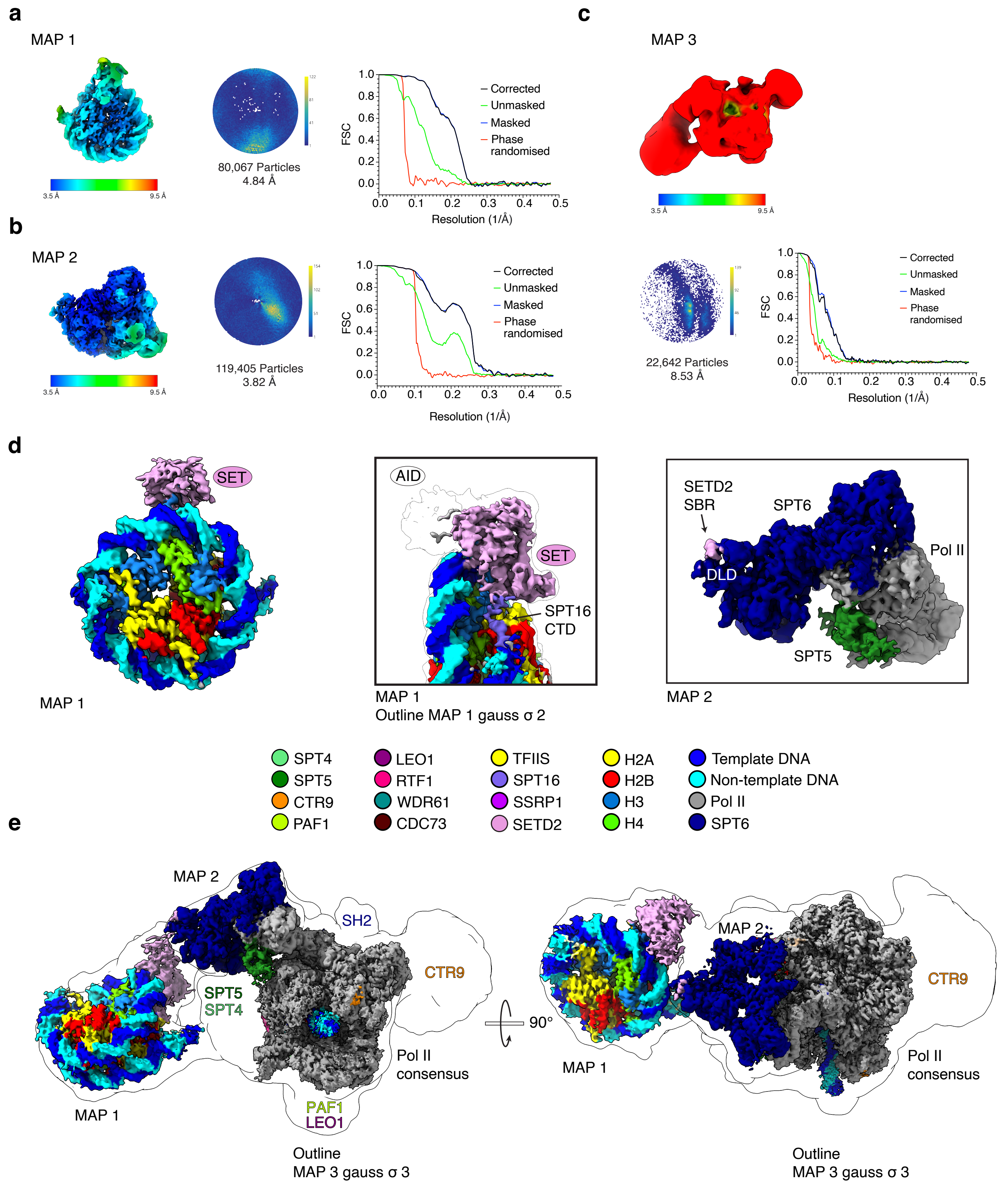

Supplementary Figure 11: Cryo-EM data metrics of State 2 complex.

a-c, Reconstructions obtained from State 2 (MAP1-3) dataset coloured by their local resolution as estimated using RELION. Total particle count, global resolution estimate using the Fourier shell correlation (FSC) = 0.143 criterion, and angular distribution plots are shown.
d, Various reconstructions highlighting data quality.
e, MAP 1, MAP 2 and Pol II consensus refinement placed within MAP 3 to show relative orientation of components.

Supplementary Figure 12

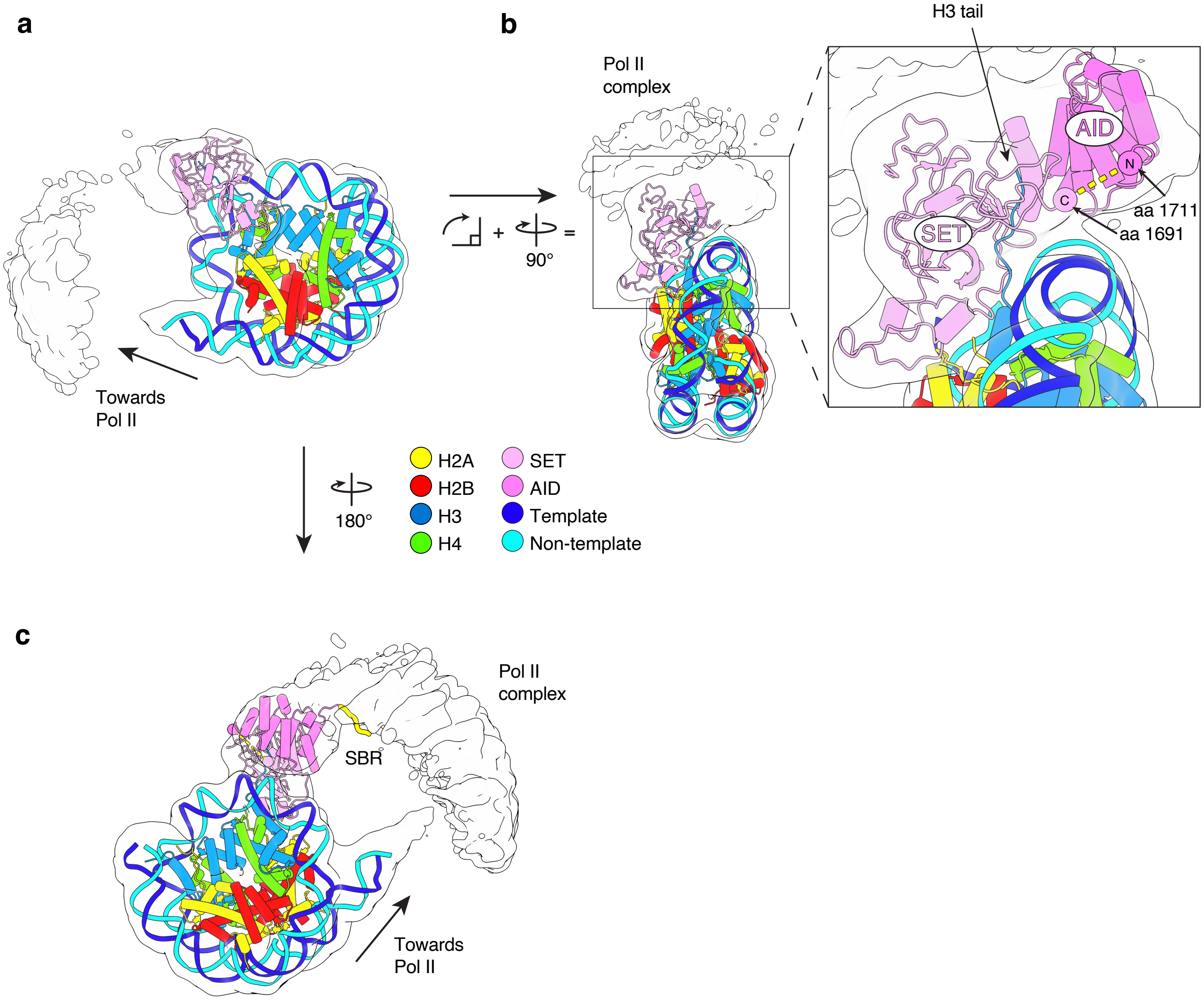

Supplementary Figure 12: SETD2 bound nucleosome in State 2 complex.

a, View of State 2 SETD2 bound nucleosome with gaussian filtered (σ = 2) cryo-EM density of MAP 1 (Supplementary Figure 11a) displayed in grey.
b, Alphafold2 model for SETD2 AID manually fitted into density and arranged to minimize distance between N and C terminal.
c, Alternate view of (a, b) with conserved residues of SBR shown as yellow tube.

Supplementary Figure 13

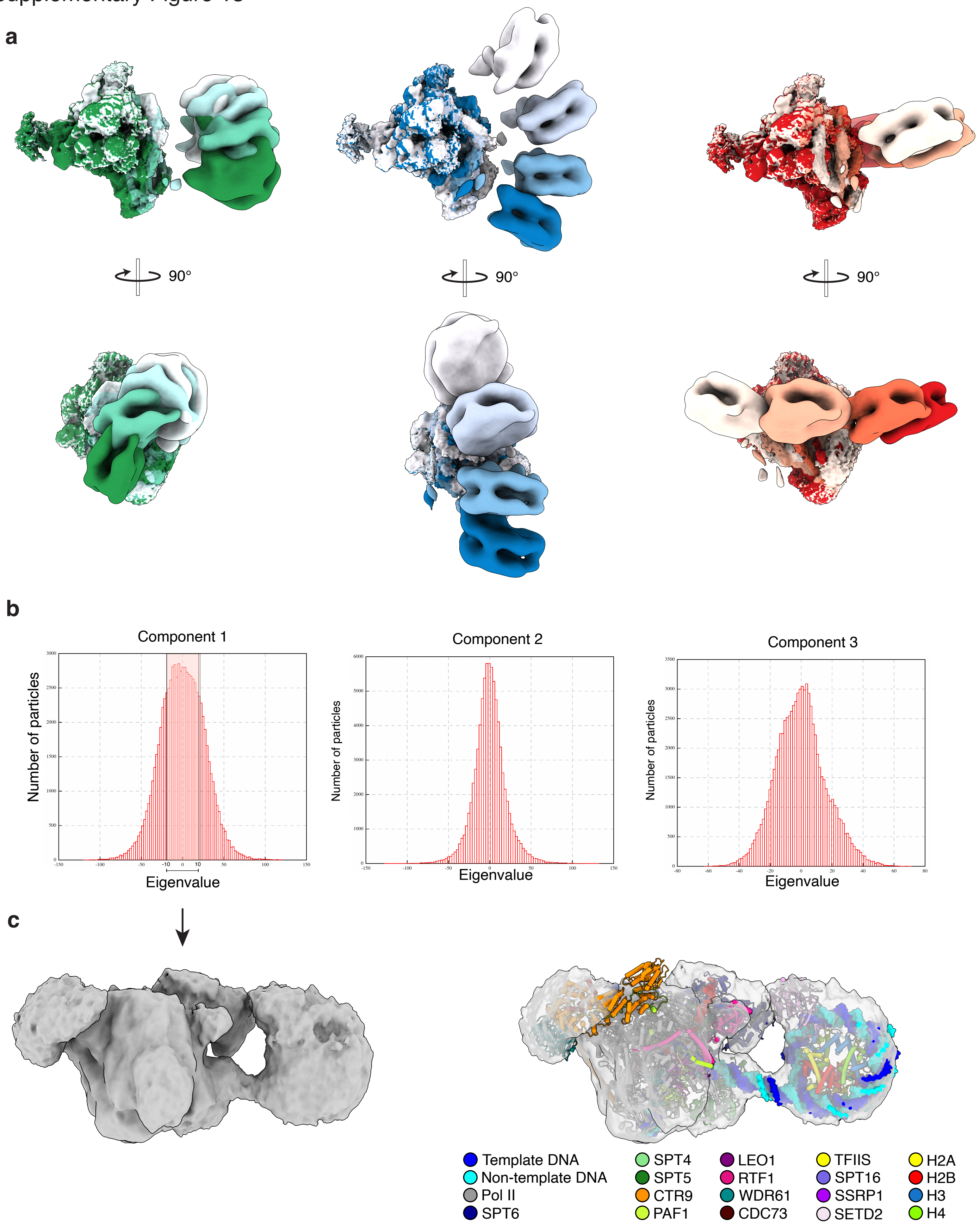

Supplementary Figure 13: Multibody refinement analysis of State 2.

a, Selected maps showing the extent of the first 3 principal motion components from multibody refinement analysis of State 2 are shown in green, blue and red respectively.
b, Eigenvalues for the first 3 principal motion components. Selected values of component 1 highlighted.
c, Refinement of selected particles from component 1 (after re-centering) with fitted model of State 2 complex.

Supplementary Figure 14

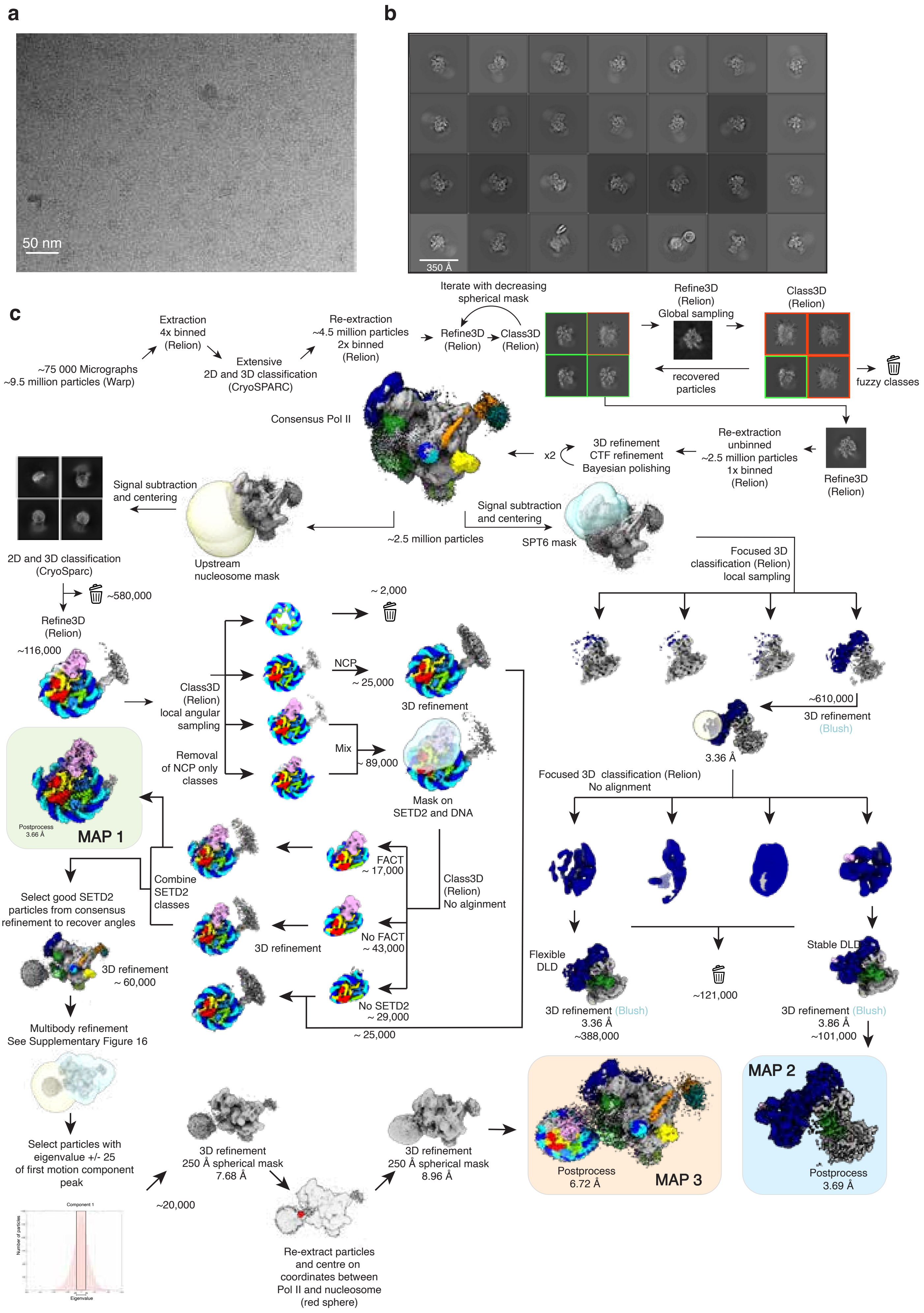

Supplementary Figure 14: Data acquisition and processing of State 3 complex.

a, Representative micrograph of the State 3 data collection with scale bar.
b, Representative 2D classes of mammalian activated elongation complex obtained during classification.
c, Processing and classification strategy employed to classify the methylation competent elongation complex from obtained micrographs. Major steps are described within the figure. Coulomb maps used to build models are highlighted.

Supplementary Figure 15

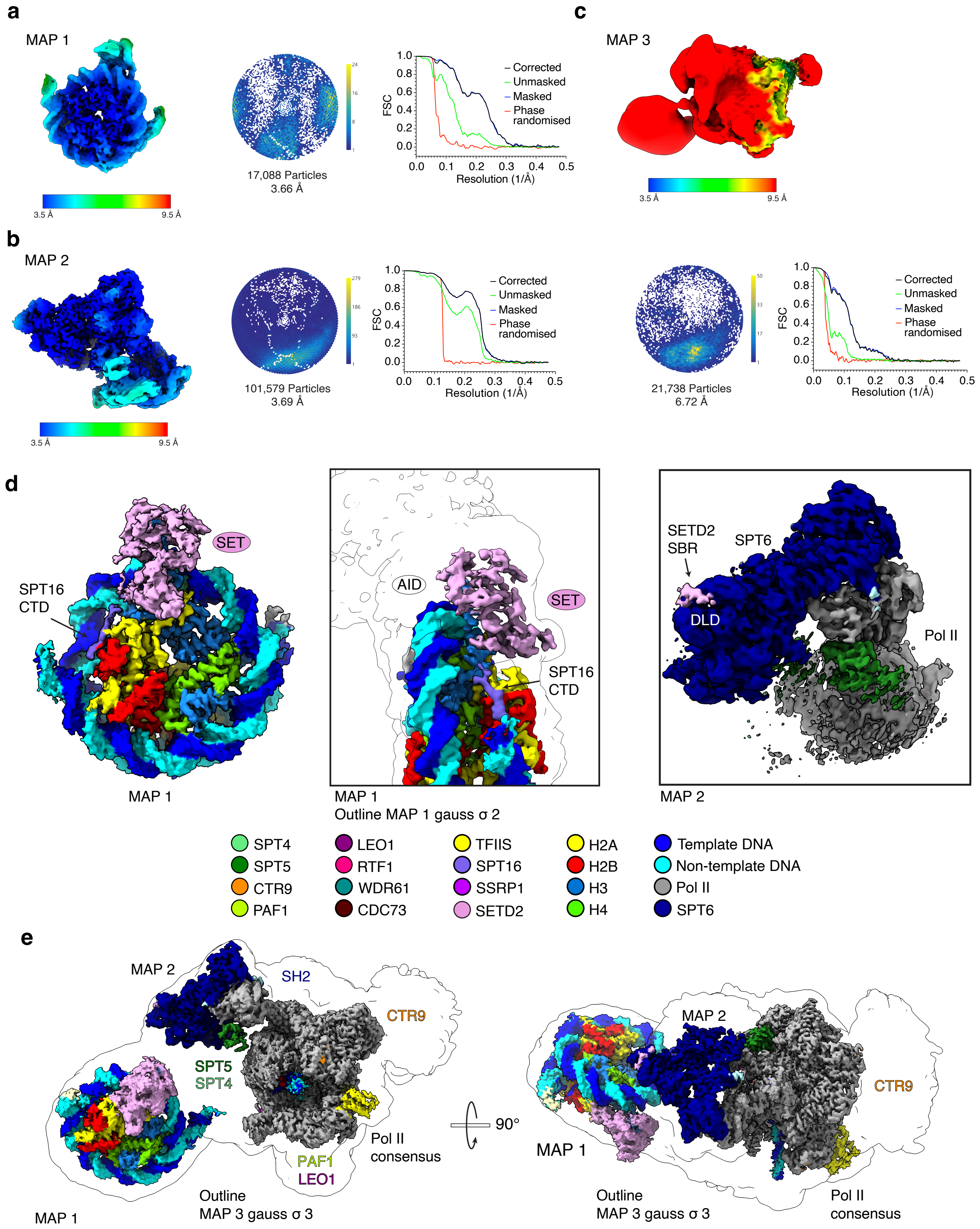

Supplementary Figure 15: Cryo-EM data metrics of State 3 complex.

a-c, Reconstructions obtained from State 3 (MAP1-3) dataset coloured by their local resolution as estimated using RELION. Total particle count, global resolution estimate using the Fourier shell correlation (FSC) = 0.143 criterion, and angular distribution plots are shown.
d, Various reconstructions highlighting data quality.
e, MAP 1, MAP 2 and Pol II consensus refinement placed within MAP 3 to show relative orientation of components.

Supplementary Figure 16

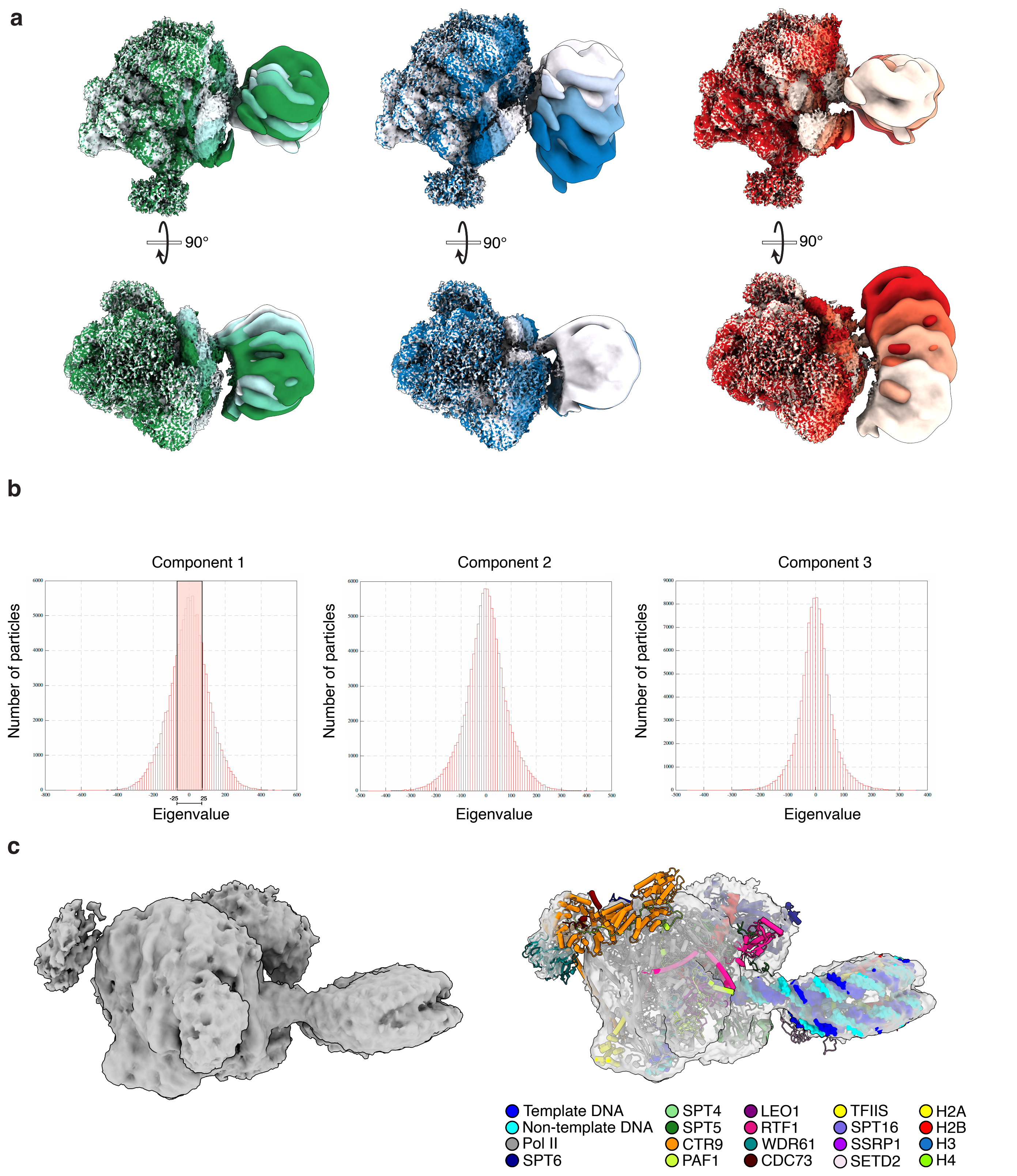

Supplementary Figure 16: Multibody refinement analysis of State 3.

a, Selected maps showing the extent of the first 3 principal motion components from multibody refinement analysis of State 3 are shown in green, blue and red respectively.
b, Eigenvalues for the first 3 principal motion components. Selected values of component 1 highlighted.
c, Refinement of selected particles (after re-centering) from component 1 with fitted model of State 3 complex.

Supplementary Figure 17

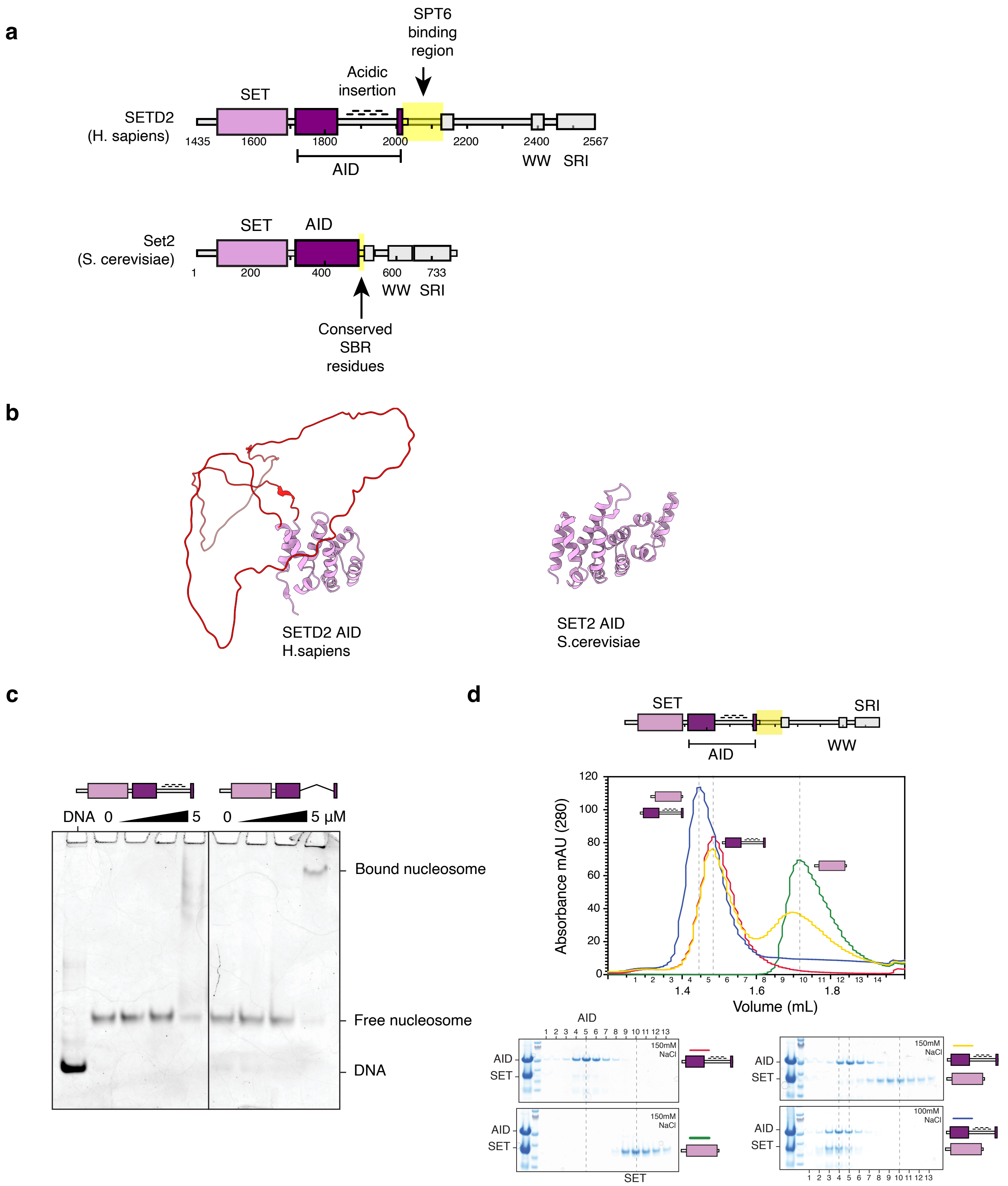

Supplementary Figure 17: Alphafold2 auto-inhibitory domain comparison and binding assays.

a, Schematic overview of the *H.sapiens* SETD2 and *S.cerevisiae* Set2 auto-inhibitory domains
b, Alphafold2 comparison of auto-inhibitory domains. Acidic insertion within the SETD2 AID colored red.
c, Electromobility shift assay of SETD2 truncation with and without acidic insertion.
d, Analytical gel filtration assay of SETD2 domain truncations. Interaction between SET domain and AID disrupted by increased ionic strength. Source data for **c,** and **d,** are provided as a Source Data file.

Supplementary Figure 18

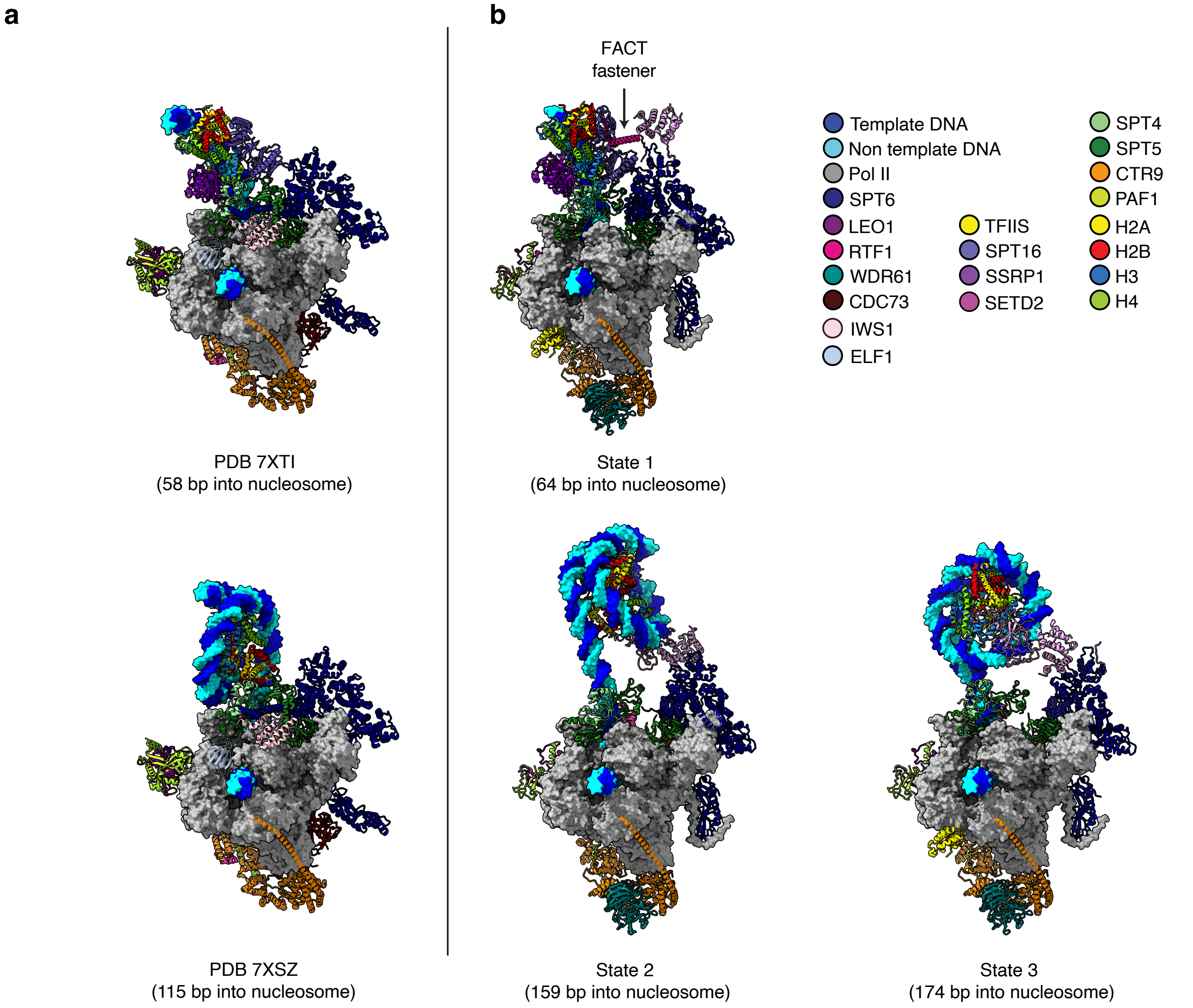

Supplementary Figure 18: Structural comparison of Pol II upstream nucleosome complexes.

a, Upstream nucleosome structures of *Komagataella pastoris* Pol II transcribed to 58 bp or 115 bp into the nucleosome 28. PDB 7XTI and 7XSZ respectively[6].
b, State 1-3 that represents 64, 159 or 174 bp of transcription into the nucleosome.

Supplementary Table 1

State 1 - RNA Pol II activated elongation complex with SETD2 and upstream hexasome

MAP3 - EMDB-54537 - PDB:9S3G

**Data collection and processing**	
Magnification	81 000x
Voltage (kV)	300
Electron exposure (e–/Å^2)	39.83
Defocus range (μm)	0.5-2.0
Pixel size (Å)	1.05
Initial particle images (no.)	1,156,702
Final particle images (no.)	112,302
Map resolution (Å)	6.4
FSC threshold	0.143
Map sharpening *B* factor (Å^2)	-87.4

	**MAP1**	**MAP2**	**MAP4**
EMDB	54538	54541	54542
Final particle images (no.)	231,990	112,302	129,213
Map resolution (Å)	3.89	3.86	3.58
FSC threshold	0.143	0.143	0.143
Map sharpening *B* factor (Å^2)	-87.6	-89.5	-36.3

Refinement and validation

Initial model used (PDB code)	6TED		
Model composition		Validation	
Non-hydrogen atoms	75,313	MolProbity score	2.14
Protein residues	8,999	Clashscore	12.36
Nucleotide residues	143	Poor rotamers (%)	1.81
Ligands	10		
B factors (Å^2)		Ramachandran plot	
Protein	109.02	Favored (%)	95.13
Nucleotide	239.49	Allowed (%)	4.53
Ligand	134.01	Disallowed (%)	0.33
R.m.s. deviations			
Bond lengths (Å)	0.009		
Bond angles (°)	1.645		

Supplementary Table 2

State 2 - RNA Pol II activated elongation complex with SETD2 bound to proximal upstream H3

**Data collection and processing**			
Magnification	81 000x		
Voltage (kV)	300		
Electron exposure (e–/Å^2)	39.83		
Defocus range (µm)	0.65-1.75		
Pixel size (Å)	1.05		
Initial particle images (no.)	746,279		
	**MAP1**	**MAP2**	**MAP3**
EMDB	51643	54247	54401
Final particle images (no.)	80,067	119,405	22,642
Map resolution (Å)	4.84	3.82	8.53
FSC threshold	0.143	0.143	0.143
Map sharpening *B* factor (Å^2)	-152.6	-60	-10
**Model refinement and validation**			
Initial model used (PDB code)	7EA8	6TED	6TED
Submitted PDB	9GW2	9RTN	9RZE
Model composition			
Non-hydrogen atoms	13,114	11,719	72,814
Protein residues	1003	1447	8,179
Nucleotide residues	250	-	372
Ligands	-	-	10
B factors (Å^2)			
Protein	94.54	86.89	58,27
Nucleotide	101.65	-	59.46
Ligand	-	-	68.23
R.m.s. deviations			
Bond lengths (Å)	0.006	0.006	0.012
Bond angles (°)	1.893	1.348	1.815
Validation			
MolProbity score	0.93	1.19	1.82
Clashscore	1.41	2.53	6.58
Poor rotamers (%)	0.71	0.00	1.61
Ramachandran plot			
Favored (%)	97.76	97.12	95.70
Allowed (%)	2.24	2.88	4.19
Disallowed (%)	0.00	0.00	0.11

Supplementary Table 3

State 3 - RNA Pol II activated elongation complex with SETD2 bound to distal upstream H3

**Data collection and processing**			
Magnification	81 000x		
Voltage (kV)	300		
Electron exposure (e–/Å^2)	39.83		
Defocus range (µm)	0.65-1.75		
Pixel size (Å)	1.05		
Initial particle images (no.)	2,499,195		
	**MAP1**	**MAP2**	**MAP3**
EMDB	54399	54400	54425
Final particle images (no.)	17,088	101,579	21,738
Map resolution (Å)	3.66	3.69	6.72
FSC threshold	0.143	0.143	0.143
Map sharpening *B* factor (Å^2)	-40	-80	-50
**Model refinement and validation**			
Initial model used (PDB code)	7EA8	6TED	6TED
Submitted PDB	9RZC	9RZD	9S0U
Model composition			
Non-hydrogen atoms	13,244	11,719	73,122
Protein residues	1003	1,447	8,260
Nucleotide residues	256	-	360
Ligands	-	-	10
B factors (Å^2)			
Protein	90.61	53.35	61,63
Nucleotide	208.86	-	73.46
Ligand	-	-	68.10
R.m.s. deviations			
Bond lengths (Å)	0.007	0.012	0.006
Bond angles (°)	1.328	1.975	1.142
Validation			
MolProbity score	1.31	0.73	1.94
Clashscore	3.37	0.17	8.70
Poor rotamers (%)	0.12	0.39	1.61
Ramachandran plot			
Favored (%)	96.95	97.12	95.55
Allowed (%)	3.05	2.88	4.32
Disallowed (%)	0.00	0.00	0.14

Supplementary References

1. Vos, S. M., Farnung, L., Linden, A., Urlaub, H. & Cramer, P. Structure of complete Pol II–DSIF–PAF–SPT6 transcription complex reveals RTF1 allosteric activation. *Nat. Struct. Mol. Biol.* **27**, 668–677 (2020).

2. Mirdita, M. et al. ColabFold: making protein folding accessible to all. *Nat. Methods* **19**, 679–682 (2022).

3. Sievers, F. & Higgins, D. G. Clustal Omega for making accurate alignments of many protein sequences. *Protein Sci. Publ. Protein Soc. 27*, 135–145 (2018).

4. Robert, X. & Gouet, P. Deciphering key features in protein structures with the new ENDscript server. *Nucleic Acids Res.* **42**, W320–W324 (2014).

5. Hornbeck, P. V. et al. PhosphoSitePlus, 2014: mutations, PTMs and recalibrations. *Nucleic Acids Res.* **43**, D512–D520 (2015).

6. Ehara, H., Kujirai, T., Shirouzu, M., Kurumizaka, H. & Sekine, S. Structural basis of nucleosome disassembly and reassembly by RNAPII elongation complex with FACT. *Science* **377**, eabp9466 (2022).
