# Supplementary material for: Molecular mechanism of co-transcriptional H3K36 methylation by SETD2
Source: Nat Commun. 2025 Oct 29;16:9565. doi: 10.1038/s41467-025-65439-y (PMC12572195; doi:10.1038/s41467-025-65439-y)
Supplement: Supplementary file 4 — Transparent Peer Review file [file 41467_2025_65439_MOESM4_ESM.pdf]

nature portfolio

Peer Review File

Molecular mechanism of co-transcriptional H3K36 methylation by SETD2

Corresponding Author: Dr James Walshe

This manuscript has been previously reviewed at another journal. This document only contains information relating to versions considered at Nature Communications.

This file contains all reviewer reports in order by version, followed by all author rebuttals in order by version.

Version 0:

Reviewer comments:

Reviewer #1

(Remarks to the Author)
I appreciate the authors for showing the cryo-EM maps for the critical regions, allowing the readers to understand their dynamic features and possibly better indicating their functions. I have no further concerns and would like to support publication of this study without further delay.

Reviewer #2

(Remarks to the Author)
I read through this interesting manuscript and the reviewers's comments. I feel that it is a high impact paper and that the authors have adequately addressed all the reviewers' comments through additional data and clarifications.

Open Access This Peer Review File is licensed under a Creative Commons Attribution 4.0 International License, which

permits use, sharing, adaptation, distribution and reproduction in any medium or format, as long as you give appropriate credit to the original author(s) and the source, provide a link to the Creative Commons license, and indicate if changes were made.
In cases where reviewers are anonymous, credit should be given to 'Anonymous Referee' and the source.
The images or other third party material in this Peer Review File are included in the article's Creative Commons license, unless indicated otherwise in a credit line to the material. If material is not included in the article's Creative Commons license and your intended use is not permitted by statutory regulation or exceeds the permitted use, you will need to obtain permission directly from the copyright holder.
To view a copy of this license, visit https://creativecommons.org/licenses/by/4.0/
